# Molecular phylogeny of Asian *Ardisia* (Myrsinoideae, Primulaceae) and their leaf-nodulated endosymbionts, *Burkholderia s*.*l*. (Burkholderiaceae)

**DOI:** 10.1371/journal.pone.0261188

**Published:** 2022-01-19

**Authors:** Chen-Jui Yang, Jer-Ming Hu

**Affiliations:** Institute of Ecology and Evolutionary Biology, National Taiwan University, Taipei, Taiwan; National Agri-Food Biotechnology Institute (NABI) Mohali, INDIA

## Abstract

The genus *Ardisia* (Myrsinoideae, Primulaceae) has 16 subgenera and over 700 accepted names, mainly distributed in tropical Asia and America. The circumscription of *Ardisia* is not well-defined and sometimes confounded with the separation of some small genera. A taxonomic revision focusing on *Ardisia* and allies is necessary. In the *Ardisia* subgenus *Crispardisia*, symbiotic association with leaf-nodule bacteria is a unique character within the genus. The endosymbionts are vertically transmitted, highly specific and highly dependent on the hosts, suggesting strict cospeciation may have occurred in the evolutionary history. In the present study, we aimed to establish a phylogenetic framework for further taxonomic revision. We also aimed to test the cospeciation hypothesis of the leaf-nodulate *Ardisia* and their endosymbiotic bacteria. Nuclear ITS and two chloroplast intergenic spaces were used to reconstruct the phylogeny of Asian *Ardisia* and relatives in Myrsinoideae, Primulaceae. The 16S-23S rRNA were used to reconstruct the bacterial symbionts’ phylogeny. To understand the evolutionary association of the *Ardisia* and symbionts, topology tests and cophylogenetic analyses were conducted. The molecular phylogeny suggested *Ardisia* is not monophyletic, unless *Sardiria*, *Hymenandra*, *Badula* and *Oncostemum* are included. The results suggest the generic limit within Myrsinoideae (Primulaceae) needs to be further revised. The subgenera *Crispardisia*, *Pimelandra*, and *Stylardisia* were supported as monophyly, while the subgenus *Bladhia* was separated into two distant clades. We proposed to divide the subgenus *Bladhia* into subgenus *Bladhia s*.*str*. and subgenus *Odontophylla*. Both of the cophylogenetic analyses and topology tests rejected strict cospeciation hypothesis between *Ardisia* hosts and symbiotic *Burkholderia*. Cophylogenetic analyses showed general phylogenetic concordance of *Ardisia* and *Burkholderia*, and cospeciation events, host-switching events and loss events were all inferred.

## Introduction

The circumscription of Primulaceae *s*.*l*. includes four subfamilies, Primuloideae, Myrsinoideae, Theophrastoideae, and Maesoideae, in the present taxonomic treatment [1,2]. Of these subfamilies, Myrsinoideae largely represents the traditional Myrsinaceae except the genus *Maesa* Forssk. The subfamily Myrsinoideae in the current classification system includes both herbaceous species with capsular fruits and woody species with fleshy and drupaceous fruits. The herbaceous species are mainly distributed in temperate regions, such as the temperate genera *Lysimachia* L. and *Cyclamen* L. On the other hand, the woody species, such as *Ardisia* Sw. and *Myrsine* L., are mainly distributed in tropical and subtropical regions. Recent phylogenetic studies suggested the herbaceous genera occupy a basal position within Myrsinoideae [3,4]. In contrast, the phylogenetic relationships within the woody Myrsinoideae are largely unresolved.

*Ardisia* is the most diverse genus, with over 700 accepted names, within the woody Myrsinoideae [5]. The genus is pantropically distributed and extends to temperate regions. The two most species-rich regions are in Southeast Asia and the Americas. The most comprehensive revision of the entire genus was by Mez in 1902 [3]. Since then, most taxonomic work on *Ardisia* was comprised of regional revision and local flora (e.g. [6–10]). In Mez’s monograph in 1902 [11], fourteen subgenera were recognized (yet not typified until Stone in 1989 [6]) mainly based on the characters of habit, leaf morphology, inflorescence position, and floral morphology. Five subgenera are restricted to New World (*Walleniopsis* Mez, *Synardisia* Mez, *Graphardisia* Mez, *Ardisia* (= *Pickeringia* (Nutt.) Mez), and *Icacorea* (Aubl.) Pax), and the other nine subgenera are distributed in the Old World. The infrageneric classification of *Ardisia* was only slightly revised since Mez. In brief, (1) *Synardisia* (Mez) Lundell was elevated to generic level [12]; (2) *Auriculardisia* Lundell and *Tetrardisia* (Mez) K. Larsen & C.M. Hu were reduced to subgenera in *Ardisia* [13,14]; and (3) a Philippine endemic subgenus, *Scherantha* B.C. Stone, was proposed [7]. In summary, *Ardisia* were classified into sixteen subgenera in the present classification. The East Asian and Southeast Asian species can be found in eleven of them, and the Neotropical species can be found in five subgenera, regardless of a few amphi-Pacific species [15].

Species of *Ardisia* are shrubs to small trees, sometimes subshrubs to herbs. In Mez’s monograph [11], *Ardisia* was distinguished from the other genera in Myrsinoidea by having free stamens and pluriseriate ovules. However, recent taxonomic works revealed these diagnostic characters were questionable and the generic limit was doubted by several other authors (e.g. [12,16–18]). Moreover, several small genera were moved back and forth from *Ardisia* since Mez’s monograph, both in Paleotropics (e.g. *Sadiria* Mez, *Afrardisia* Mez, *Tetradisia* Mez, *Hymenandra* A. DC., and *Antistrophe*) and Neotropics (e.g. *Icacorea* Aubl., *Auriculardisia* Lundell, *Graphardisia* (Mez) Lundell, and *Synardisia* (Mez) Lundell). Some small genera were combined with *Ardisia* and sometimes were treated as subgeneric rank. The taxonomic uncertainty indicated that the generic limit of *Ardisia* and allies is ambiguous. The generic delimitation of *Ardisia* remains a challenge and a widely accepted description of the genus is still unavailable, as is true of many other Myrsinoideae genera.

To conduct a phylogenetic analysis is crucial in determining the circumscription of *Ardisia* and infrageneric classification. Ku and Hu conducted a phylogenetic analysis including 36 *Ardisia* taxa belonging to nine subgenera, suggesting that the present subgeneric classification can only partially reflect the phylogeny [19]. More recent studies suggested the genus *Ardisia* is not monophyletic [20,21]. Zhou and colleagues conducted phylogenetic studies based on nuclear ribosomal internal transcribed spacers (nrITS) and six plastid markers including 25 *Ardisia* species, and their results suggested that the genus *Amblyanthus* A. DC. and *Amblyanthopsis* Mez are nested within the *Ardisia* clade and sister to the subgenus *Crispardisia* Mez clade [21]. Julius and colleagues have accomplished the most comprehensive phylogenetic study on *Ardisia* and allies to date [20], although their phylogeny was only based on nrITS. The results also showed the *Ardisia* clade was not monophyletic. Several allied genera were nested within the *Ardisia* clade, including *Labisia* Lindl., *Badula* Juss., *Discocalyx* Mez, *Fittingia* Mez, *Hymenandra* (A. DC.) Spach, *Oncostemum* A. Juss., *Sadiria* Mez, and *Systellantha* B.C. Stone. They also noticed that the subgeneric classification was only partially reflecting the phylogenetic relationships. Nevertheless, the relationships of the genera and subgenera were poorly resolved in the previous studies.

Among the *Ardisia* subgenera, *Crispardisia* can be easily recognized by the swollen leaf nodules localizing on the incisions of the crenations on the leaf margin, along the entire margin, or rarely within the tips of the dentation. The leaf nodule is a structure harboring symbiotic bacteria, which is potentially beneficial by synthesizing defensive secondary metabolites or growth factors [22,23]. Similar bacterial leaf nodule symbiosis also occurs in Rubiaceae, Dioscoreaceae, and possibly in Styracaceae, which is a rare phenomenon in angiosperms [23,24]. Only one single *Burkholderia s*.*l*. species can be found within the leaf nodule within an individual in *Ardisia* [19]. However, the symbiont-host specificity at species level has not been validated thoroughly. In other words, the question of whether a single bacterial species can only be found in a single given species of *Ardisia* has not been well studied, and vice versa. The bacterial symbionts are also present in the shoot apex in *Ardisia*, and are able to infect every new leaf and the emerging floral buds, including carpels and embryos [24]. Thus, the leaf nodule symbiosis in *Ardisia* is regarded as a vertically transmitted, closed, and cyclically inherited system. The vertical transmission of a symbiont lineage within host lineages often leads to phylogenetic congruence between the symbiont and the host (e.g. *Helicobacter pylori* in humans [25]). Nevertheless, the cophylogenetic studies suggest host shifts in speciation occurred at least two or three times in Rubiaceae [26], which has a similar symbiotic system with *Ardisia*. Topological incongruence between the hosts and symbionts was also found in *Ardisia* [19], but the incongruence occurred only within certain clades. The strict cospeciation may not occur in the *Ardisia*-*Burkholderia* association, while the overall evolutionary scenario remains unclear.

The aims of this study were to [1] reconstruct the phylogenetic relationships of the *Ardisia* clade, especially focusing on the subgenus *Crispardisia*; and [2] understand the cophylogenetic relationships of the leaf-nodulated *Ardisia* (i.e. subgenus *Crispardisia*) and their symbionts. Here, we used nrITS and two plastid intergenic spacers (IGS), *psbA-trnH* and *rpl32-trnL* as molecular markers to construct the *Ardisia* phylogeny. The nrITS and *psbA*-*trnH* were widely used as molecular markers with moderate to high discrimination [19,27,28]. To further increase the resolution of phylogenetic analysis, *rpl32*-*trnL* was chosen in the present study based on the study on plastid genome of *Ardisia polysticta* Miq., which is one of the most variable regions across the plastid genome in *Ardisia*-*Sesamum* L. and *Ardisia*-*Panax* L. comparisons [29]. The previous study suggested that 16S rRNA, ITS, and partial 23S rRNA (16S-23S rRNA) region is a powerful molecular marker for resolving the *Burkholderia* phylogeny on a species level [19], and thus the region was used for constructing the symbiotic bacteria phylogeny. In this study, the results suggested *Ardisia* is not monophyletic, and the generic limit within Myrsinoideae (Primulaceae) needs to be further revised. In addition, the cophylogenetic analyses showed general phylogenetic concordance of leaf-nodulate *Ardisia* and their symbionts, suggesting host-switching events and loss events also played a role in the evolution of the leaf nodule symbiosis in *Ardisia*.

## Materials and methods

### Ethics statement

This study did not require any special permits because all collecting was performed by researchers located at institutes with the permits required, including FOF (Faculty of Forest Science) in Laos, Guangxi Institute of Botany, Chinese Academy of Sciences in China, Institute of Ecology and Evolutionary Biology in Taiwan, National Institute for Environmental Studies in Japan, Prince of Songkla University in Thailand, VAFS (Forest Science Institute of Vietnam) in Vietnam, and VNM (Institute of Tropical Biology) in Vietnam (acronyms followed [30]). Three samples are harbored materials deposited in the Dr. Cecilia Koo Botanic Conservation Center (KBCC) in Taiwan.

### Taxa sampling, DNA extraction, PCR amplification, and sequencing

Fresh materials were collected from the fields in China, Japan, Laos, Taiwan, Thailand, and Vietnam from 2012 to 2018. For each specimen, fresh leaves were collected and immediately placed into sealed plastic bags containing >10 g silica gel for dehydration. Vouchers of freshly collected material were deposited at herbaria of FOF, HNL, KYO, MAK, TAI, TI, and VNM (acronyms followed [30]). Several herbarium materials collected from TAI and TAIF were also used. Species names, voucher information and GenBank accession numbers of sequences are listed in S1 Table.

Silica-dried materials and herbarium materials were used to extract total DNA with CTAB method [31]. Leaf tissue with at least one or two leaf nodules, if present, was used. For rare or herbarium materials, RNase treatment was omitted.

The sequences of nuclear ribosomal internal transcribed spacer (nrITS) and two plastid intergenic spacers (IGS), *psbA-trnH* and *rpl32-trnL*, were used for *Ardisia* phylogenetic analyses. For the *Ardisia* symbionts, the sequences of 16S rRNA (or partial 16S rRNA), ITS, and partial 23S rRNA (16S-23S rRNA) were amplified with three to five primer pairs. For the bacterium with two types of *rrn* operons [19], only type 2A was sequenced with specific primer pairs and used in the following phylogenetic analyses (S2 Table). All the primer sequences and annealing temperature used in this investigation are listed in S2 Table. PCR condition was described in Ku and Hu [19]. All PCR products were purified and sequenced directly.

For samples that failed to obtain clear sequence signals, the PCR products were cloned using pGEM-easy Easy Vector System (Promega, Madison, WI, USA) and *Escherichia coli* DH5α competent cells (Yeastern Biotech, Taipei, Taiwan). The plasmids with correct insertion size were extracted and sequenced. At least three clones were sequenced for each sample.

### Sequence alignment and phylogenetic analyses

Raw sequences were visualized, assembled, and edited using Sequencher v5.2.4 [32]. The chromatogram of the raw sequence was examined by naked eye. For the nrITS, some nucleotides were found with double peaks, and those sites were coded as ambiguity character followed by IUPAC nomenclature. The ambiguity characters were coded as missing data in the subsequent analyses. No ambiguity character was found and coded in plastid datasets and bacterial 16S rRNA and 16S-23S rRNA datasets. DNA sequences were aligned using the online web tool, MAFFT v7 [33,34]. Different datasets (nrITS, *psbA-trnH*, *rpl32-trnL*, and bacterial 16S rRNA and 16S-23S rRNA) were aligned independently. We trimmed the ends of the resulting alignments and minimized the amount of terminal missing data, except for sequences from GenBank having a shorter sequence. To reconstruct the relationship of *Ardisia* and allies in Myrsinoideae, only nrITS datasets were used because nrITS is currently the most accessible genetic marker on GenBank. Sequences downloaded from GenBank were incorporated in the combined dataset if the vouchers were annotated. Unavailable sequences in combined dataset were coded as missing data. Outgroups of *Ardisia* and allies for phylogenetic reconstruction were selected based on the phylogenies of Källersjö et al. [35] and Yan et al. [36]. Sequences were edited and combined in BioEdit v7.2.5 [37].

To test the monophyly of the *Ardisia* symbionts within *Burkholderia s*.*l*., 16S rRNA sequences were aligned with sequences of other *Burkholderia s*.*l*. species (including *Caballeronia*, *Mycetohabitans*, *Paraburkholderia* and *Trinickia* species) downloaded from GenBank (S3 Table).

Best-fit DNA substitution models were selected by the Akaike information criterion (AIC) implemented in jModelTest2 v2.1.6 [38] on XSEDE from the CIPRES Science Gateway for each data partition [39]. The substitution model of the sequences was set to the most comparable model, if the suggested model is not available in the software. Phylogenies were reconstructed using the maximum likelihood (ML) method in RAxML-HPC2 v8.2.12 [40] on XSEDE of the CIPRES [39]. ML analyses were analyzed with 1000 bootstrap resampling with the gamma model of rate heterogeneity. Bayesian inference (BI) analyses were performed using MrBayes v3.2.6 [41] through XSEDE implemented on the CIPRES [39]. The Markov Chain Monte Carlo analysis was performed with four chains run for 10 million generations, or stopped when average standard deviation <0.01, sampling every 1000 generations. The first 25% of trees were discarded as burn-in, and a majority-rule consensus tree was generated from the remaining trees.

### Cophylogenetic analyses

The taxa-reduced datasets with leaf-nodulated *Ardisia* and their symbionts were used for cophylogenetic analyses. *Ardisia collinsiae* H.R. Fletcher ‘Hu3034’ was used as an outgroup for the host phylogeny, and *B*. *udeis* LMG27134 was used as an outgroup, based on the 16S rRNA phylogeny, for the symbiont phylogeny. Nuclear ITS, *psbA-trnH*, and *rpl32-trnL* were used for host phylogeny reconstruction, and 16S-23S rRNA was used for symbiont phylogeny reconstruction.

The overall congruence between the host and symbiont phylogenies were tested, i.e. topology or distance-based methods. To test the overall topological congruence between the phylogenies of *Ardisia* and the symbiont, both SH tests and KH tests were performed in PAUP* v4.0a166 [42–44]. We used both the *Ardisia* dataset and symbiont dataset to determine whether the ML tree of symbionts is as good as the *Ardisia* ML tree (H0).

TreeMap and JANE v4 were used for constructing the tanglegram for hosts and symbionts [45,46]. The pairwise distance correlation test of topological congruence was performed in TreeMap, and the event-based method cophylogenetic analyses were performed in JANE. In addition, JANE was used to test whether the cost of a historical reconstruction is significantly lower than expected by chance. The pseudo-random sample of minimal overall costs was generated from a null distribution of 100 times randomizing the interactions of the tips of the two phylogenies (random tip mapping, or RTM) and the branching order of the dependent tree (random parasite tree, or RPT). To take into consideration the phylogenetic uncertainty of the host, an additional analysis was performed [47]. An observed distribution of the total costs from 100 *Ardisia* ML bootstrap trees was obtained. In addition, the total costs of the inferred historical reconstruction of the pseudo-sampling bootstrap trees are equal to those expected by chance (RTM and RPT), as null hypothesis, was also tested with the independent samples and unequal variances t-tests. The Shapiro-Wilk tests were used to test the normality assumption for the t-tests, using the Statistics Kingdom web calculator [48]. The t-tests were calculated in “Microsoft Excel (2016)”.

## Results

### Phylogenetic reconstruction

**Fig 1:**
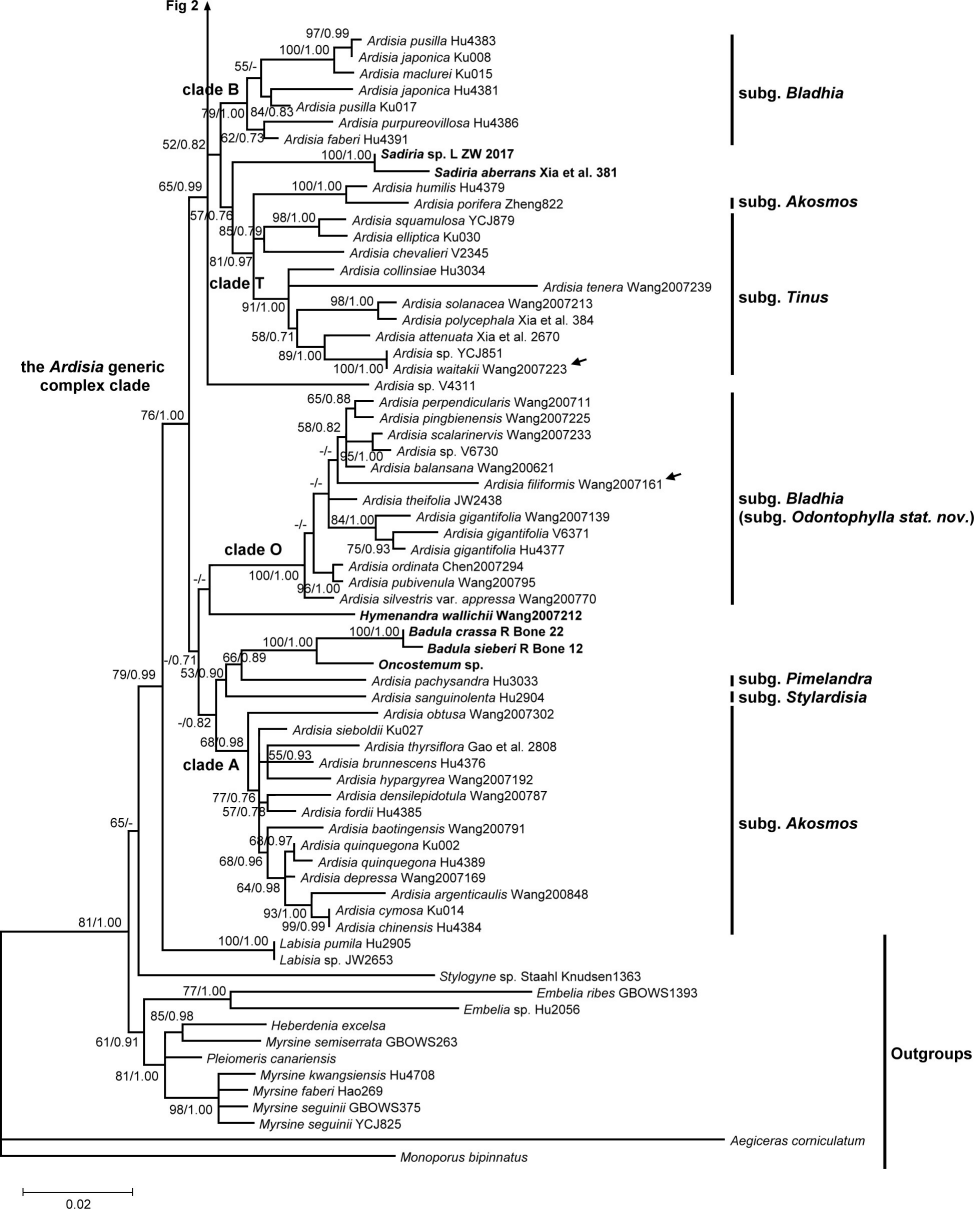
Phylogeny of *Ardisia* and allies inferred using maximum likelihood (ML) based on nuclear ITS sequences. Lower half of the tree. Numbers at branches are ML bootstrap support (ML BP) values and Bayesian posterior probabilities (BI). ML bootstrap support values are indicated only if at least 50, and the Bayesian posterior probabilities are indicated only if at least 0.7. Alternative branching between the two different analyses is showed as no support at the branch. Non-*Ardisia* species nested within the *Ardisia* generic complex are shown in boldface. Arrows indicate the presumed misidentification. Collection numbers (or other voucher information) are labeled after species names.

**Fig 2:**
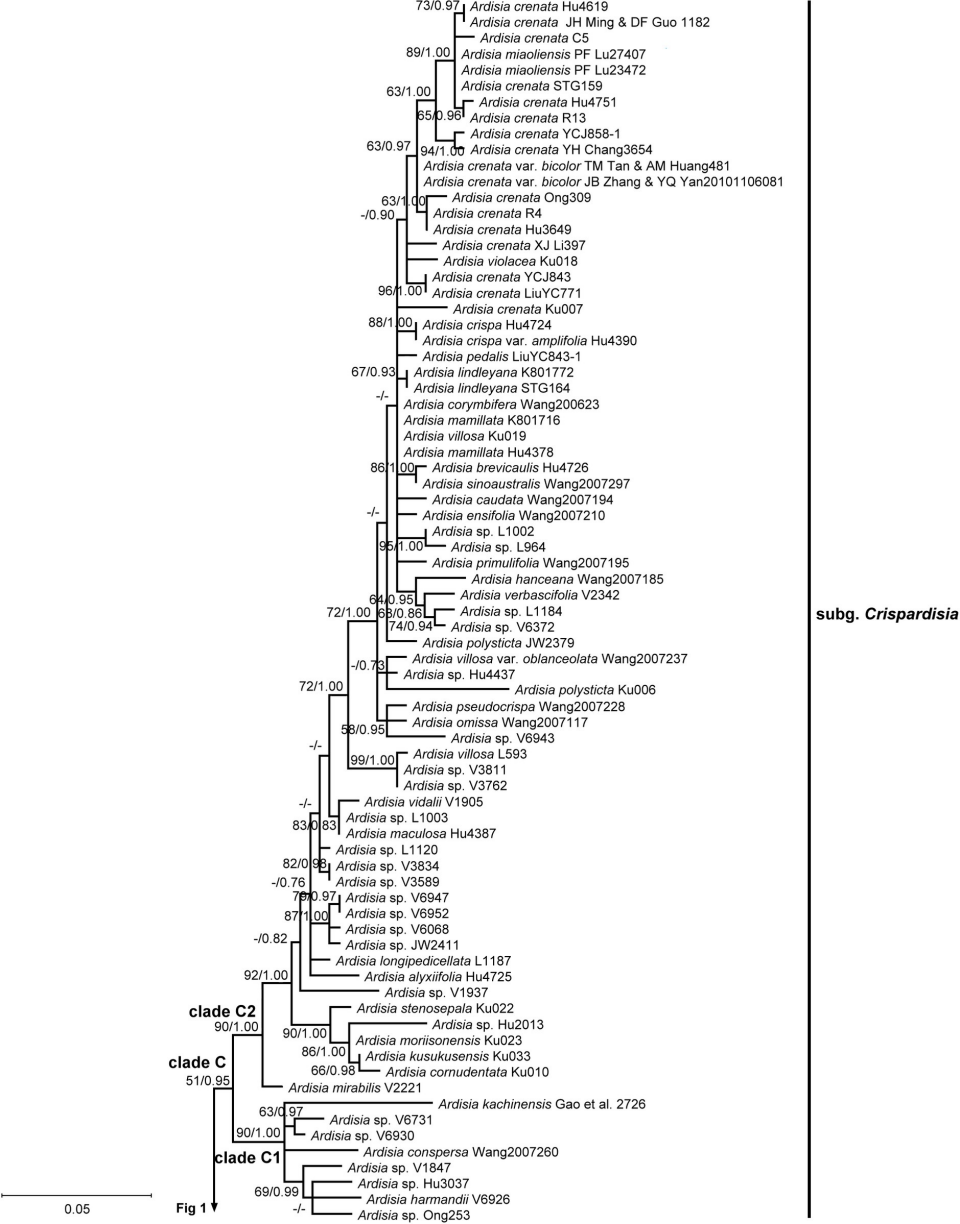
Phylogeny of *Ardisia* and allies inferred using maximum likelihood (ML) based on nuclear ITS sequences. Upper half of the tree. Numbers at branches are ML bootstrap support (ML BP) values and Bayesian posterior probabilities (BI). ML bootstrap support values are indicated only if at least 50, and the Bayesian posterior probabilities are indicated only if at least 0.7. Alternative branching between the two different analyses is showed as no support at the branch. Non-*Ardisia* species nested within the *Ardisia* generic complex are shown in boldface. Arrows indicate the presumed misidentification. Collection numbers (or other voucher information) are labeled after species names.

A total of 146 nrITS sequences were used. The nrITS dataset comprised 700 bp, including 216 parsimony-informative (PI) characters. The GTR+I+G model was selected for nrITS dataset. The genera *Aegiceras* Gaertn. and *Monoporus* A. DC. were used as the outgroups in the nrITS tree, and other Myrsinaceae members formed a monophyletic group with medium support (Figs 1 and 2). The current study was grouped into two major clades.The first clade is comprised of the genera *Embelia* Burm. f., *Myrsine* (including *Rapanea* Aubl.), *Pleiomeris* A. DC., and *Heberdenia* Banks ex A. DC., yet was weakly supported (ML BP = 61%, BI = 0.91). This clade showed *Embelia* was monophyletic while *Myrsine* was not. *Heberdenia excelsa* Banks ex DC. and *Pleiomeris canariensis* (Willd.) A. DC. were nested within the *Myrsine* clade.

The second clade included *Stylogyne* A. DC., *Labisia* Lindl., *Ardisia*, *Hymenandra*, *Badula* Juss., *Oncostemum* A. Juss., and *Sadiria* Mez. *Stylogyne* was the most basal group of this clade, though the relationship was poorly supported. The genus *Labisia* was sister to the *Ardisia* generic complex clade in the present phylogeny, with moderate support (ML BP = 79%, BI = 0.99). The genera *Hymenandra*, *Badula*, *Oncostemon*, and *Sadiria* were all nested within *Ardisia*, together forming a monophyletic group (named as the *Ardisia* generic complex here, Figs 1 and 2). In other words, the genus *Ardisia* is not monophyletic in our nrITS tree.

**Fig 3:**
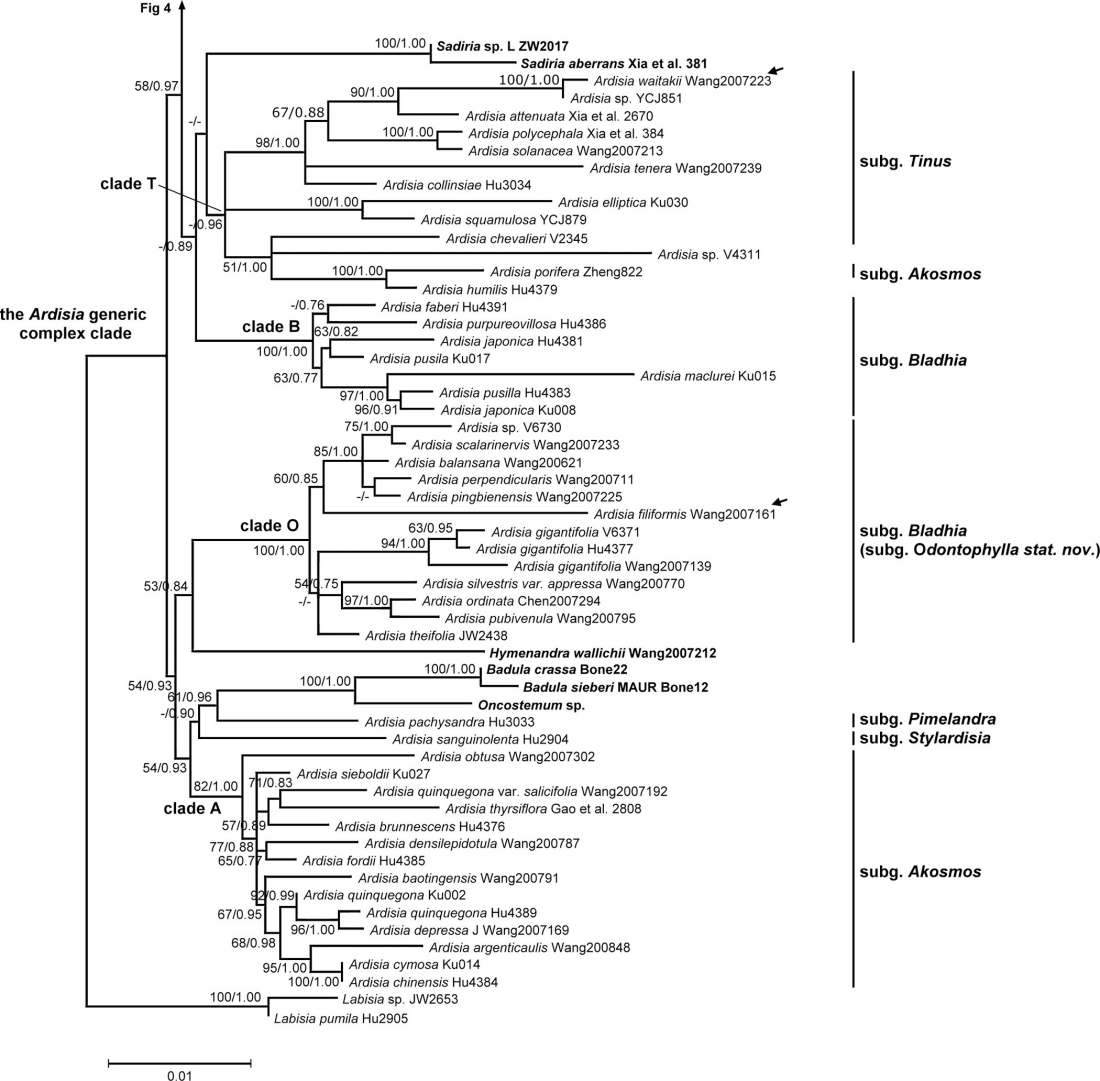
Phylogeny of *Ardisia* and allies inferred using maximum likelihood (ML) based on the combined dataset. Lower half of the tree. Combined dataset including nuclear ITS and two plastid intergenic spacer (*psbA-trnH* and *rpl32-trnL*) sequences. Numbers at branches are ML bootstrap support values and Bayesian posterior probabilities. ML bootstrap support values are indicated only if at least 50, and the Bayesian posterior probabilities are indicated only if at least 0.7. Alternative branching between the two different analyses was showed as no support at the branch. Non-*Ardisia* species nested within the *Ardisia* generic complex are shown in boldface. Arrows indicate the presumed misidentification. Collection numbers are labeled after species names.

**Fig 4:**
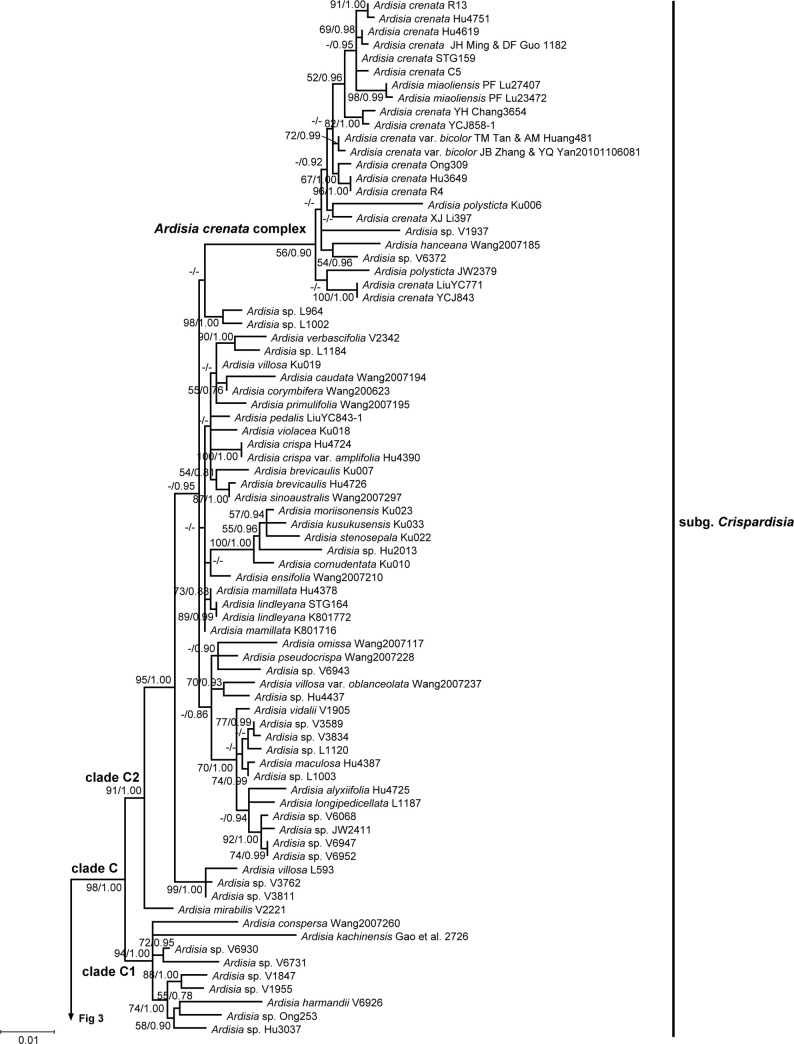
Phylogeny of *Ardisia* and allies inferred using maximum likelihood (ML) based on the combined dataset. Upper half of the tree. Combined dataset including nuclear ITS and two plastid intergenic spacer (*psbA-trnH* and *rpl32-trnL*) sequences. Numbers at branches are ML bootstrap support values and Bayesian posterior probabilities. ML bootstrap support values are indicated only if at least 50, and the Bayesian posterior probabilities are indicated only if at least 0.7. Alternative branching between the two different analyses was showed as no support at the branch. Non-*Ardisia* species nested within the *Ardisia* generic complex are shown in boldface. Arrows indicate the presumed misidentification. Collection numbers are labeled after species names.

The combined dataset comprised 135 concatenated sequences with 2221 bp, including 306 PI characters. Plastid *psbA-trnH* and *rpl32-trnL* comprised 590 and 937 bp, respectively. The best-fit models were TPM1uf+I+G for the combined dataset, TPM1uf+G for *psbA-trnH* dataset, and TPM1uf+I+G for *rpl32-trnL* dataset. The topology of combined datasets analyses is generally concordant with nrITS tree (Figs 1–4). The phylogeny showed more resolved tree topologies with higher support values in most clades, so we used the phylogenetic tree of concatenated datasets for the subsequent analyses and discussion within the *Ardisia* generic complex clade. The genus *Labisia* was used as outgroup to construct the combined dataset phylogeny.

To test the phylogenetic affinity of *Labisia* and *Ardisia*, we also reconstructed the phylogeny including *Embelia*, *Tapeinosperma* Hook. f., *Parathesis* (A. DC.) Hook. f., and *Elingamita* G.T.S. Baylis, which have complete plastid genomes on GenBank but lack nrITS sequences, with combined plastid *psbA-trnH* and *rpl32-trnL* IGS sequences (S1 Fig). *Labisia*, *Tapeinosperma*, and the *Ardisia* generic complex clade formed an unresolved tritomy in our plastid phylogeny. The results showed *Labisia* and *Tapeinosperma* was independent from the *Ardisia* generic complex, while the relationship of *Tapeinosperma*, *Labisia*, and the *Ardisia* generic complex was unresolved.

### Phylogenetic relationship of the *Ardisia* generic complex clade

Three sequences retrieved from GenBank were presumed to be misplaced or misidentified. (1) The sequence of *Ardisia filiformis* E. Walker voucher ‘Wang2007161’ (accession: MF926196.1 and MF926070.1, direct submitted) was surprisingly grouped within the subgenus *Bladhia* (Thunb.) Mez clade (clade B) in our phylogenies (Figs 1 and 3, marked with arrows). However, *A*. *filiformis* fits within the subgenus *Crispardisia* morphologically and is distinct from *Bladhia*. Three other *psbA-trnH* sequences of *A*. *filiformis* were found in GenBank (accession: MF037963.1, MF037967.1 and MF037970.1, three sequences are 100% identical), and their genetic distance suggests they are closely related to species in *Crispardisia* rather than those in *Bladhia*. Thus, the sequences from voucher Wang2007161 were probably misplaced or misidentified. (2) In clade T, species that labelled as ‘*Ardisia* sp. YCJ851’ of our own collection (accession: MW414762, MW456399, and MW456488) was sister to *A*. *waitakii* voucher ‘Wang 2007223’ (accession: MF926230.1 and MF926103.1, direct submitted), and the two sequences had only one base difference (Figs 1 and 3, marked with arrows). After careful examination, *Ardisia* sp. ‘YCJ851’, having glabrous leaves, axillary subumbel inflorescence, and calyx lobes distinctly imbricate at anthesis, clearly belongs to the subgenus *Tinus* Mez. Thus, we doubted that the *A*. *waitakii* vourcher ‘Wang 2007223’ was misidentified. (3) Another sequence retrieved from GenBank, *Ardisia dasyrhizomatica* C.Y. Wu & C. Chen voucher ‘Wang Jun 2007212’ (accession: MF926193.1 and MF926065.1, directly submitted), was misidentified and should be *Hymenandra wallichii* A. DC., according to Huang et al. [49].

Four non-*Ardisia* genera (i.e. *Hymmenandra*, *Oncostemum*, *Badula*, and *Sadiria*) were nested within the *Ardisia* generic complex clade. *Hymmenandra wallichii* was sister to clade O. The Madagascar genus *Oncostemum* and the Mascarene genus *Badula* were sister groups and together sister to *Ardisia pachysandra* (Wall.) Mez (subg. *Pimelandra* (A. DC.) Mez). *Sadiria* was also nested within the *Ardisia* generic complex clade. The phylogeny showed *Sadiria* was close to clade T and clade B, but the relationship was not well supported.

Clade A comprised all subgenus *Akosmos* Mez members except for *A*. *porifera* E. Walker, which was nested within clade T. The subgenera *Pimelandra* and *Stylardisia* Mez, both with only one species in our sampling, were not nested within other subgenera. Clade O comprised of all species of subgenus *Bladhia* section *Odontophylla* Y.P. Yang. However, species of subgenus *Bladhia* section *Bladhia* were all in the clade B, which is distinct from clade O. Clade T is composed of *Ardisia* sp. ‘V4311’, *A*. *humilis* Vahl, *A*. *porifera*, and species of subgenus *Tinus*. However, *Ardisia* sp. ‘V4311’, clade T, and clade C formed an unresolved tritomy in nrITS tree. Clade C represents the subgenus *Crispardisia*, and this clade can further be divided into two strongly supported subclades, C1 and C2. The *A*. *crenata* Sims complex, including *A*. *crenata*, *A*. *crenata* var. *bicolor* (Walker) C.Y.Wu & C.Chen, *A*. *hanceana* Mez, *A*. *miaoliensis* S.Y. Lu, and *A*. *polysticta*, was identified in clade C2.

### Phylogenetics analyses of the leaf-nodulated symbionts associated with *Ardisia*

Symbionts were successfully detected via PCR method in most of our sampling materials of the leaf-nodulated *Ardisia* (i.e. subgenus *Crispardisia*), suggesting bacterial symbionts are present in most *Ardisia* species of subgenus *Crispardisia*. Very few individuals failed to amplify bacterial DNA from leaf tissue, possibly due to the poor DNA quality and quantity.

The 16S rRNA dataset comprised of 111 sequences, including 65 *Ardisia* symbionts, with 1417 bp after alignment. The preferred model was GTR+I+G. Our 16S rRNA phylogeny of *Burkholderia s*.*l*. suggested the *Ardisia*-associated symbionts are monophyletic (S2 Fig). The *Ardisia* symbiont clade was sister to the clade of *B*. *udeis* and *B*. *sordidicola*, both consisting of environmental bacteria [50,51]. To assess the cophylogeny of the leaf-nodulated *Ardisia* and symbionts, 65 hosts and their corresponding symbionts sequences were analyzed. The combined dataset was used for the *Ardisia* host phylogeny. A total of 2903 bp of the 16S-23S rRNA dataset was used to construct a more resolved phylogeny for the symbionts (S3 Fig). TIM3+G was selected as the best-fit evolutionary model for the dataset.

**Fig 5:**
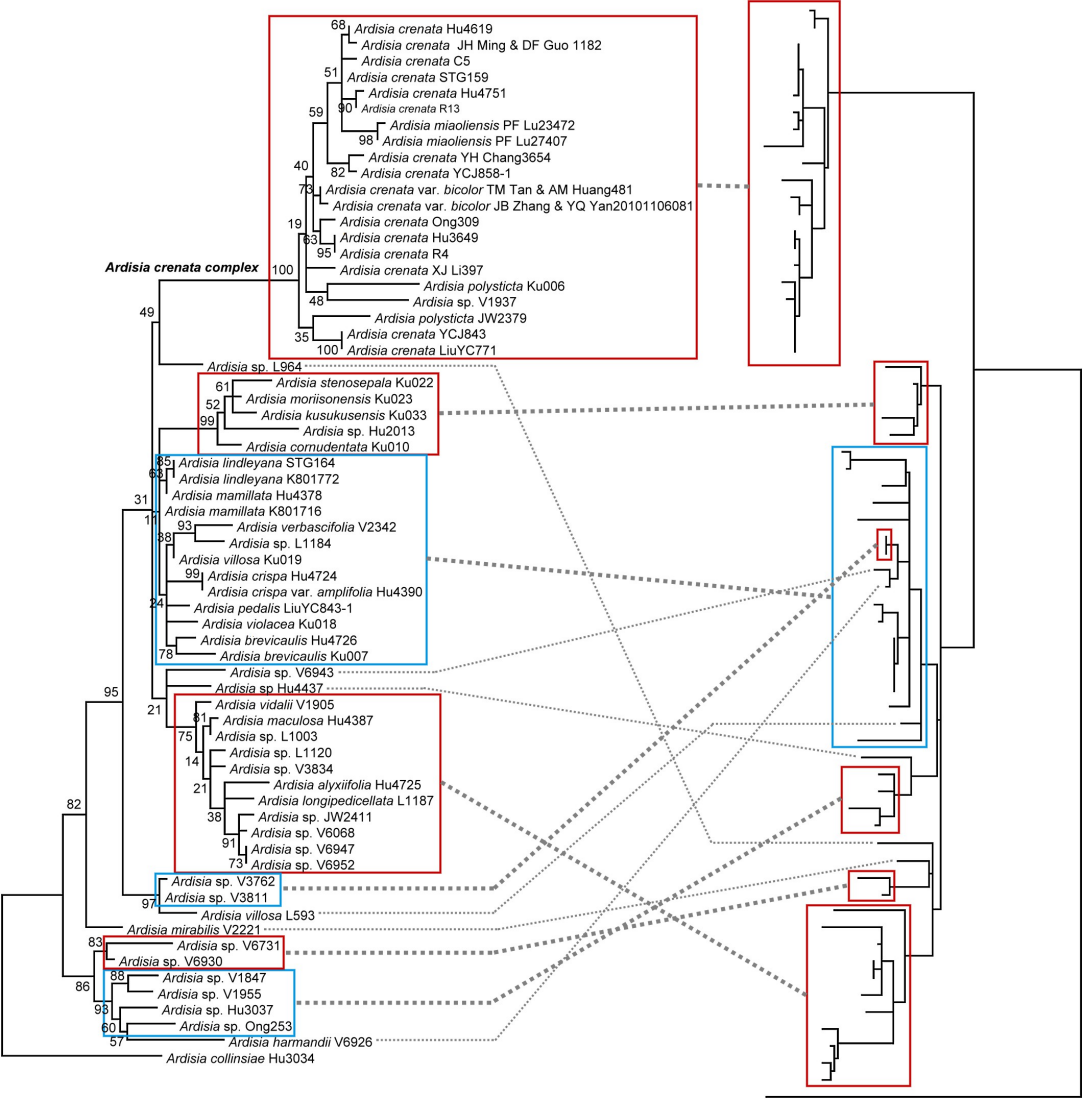
Tanglegram for *Ardisia* and their symbiotic bacteria. Symbiont species or clades (subtrees) are connected to their host by a gray line. Maximum likelihood trees based on the nrITS and plastid sequences (*psbA-trnH* and *rpl32-trnL*) for the host (left) and the 16S-23S rRNA of their symbiotic bacteria (right). Red box indicates monophyletic clade, and blue box indicates non-monophyletic clade. Numbers in the left tree are ML bootstrap support values. Collection numbers were labeled after species names.

To compare the phylogenies of *Ardisia* hosts and the associated bacteria, two different approaches were used, topology tests and cophylogenetic analyses. Both topology tests, K-H test and S-H test, rejected the null hypothesis (*P*<0.05, both host phylogeny and symbiont phylogeny are equally good), suggesting a strict cospeciation in the relationship is unlikely. Both host and symbiont phylogenies showed a large proportion of phylogenetic uncertainty, including unresolved polytomies and low-supported nodes. To visualize the cophylogenetic relationships, we constructed a tanglegram, mapping *Ardisia* hosts and symbionts by species or subtrees on the phylogenies (Fig 5). The tanglegram showed at least five or six corresponding subtrees, while the relationship within these subtrees were mostly incongruent between the two phylogenies. On the other hand, at least three clearly host switching events were discovered. *Ardisia* sp. ‘Hu4437’, *A*. *harmandii* ‘V6926’, and *Ardisia* sp. ‘L964’ were obviously in different positions between the host tree and the symbiont tree. Other phylogenetic position discordance in the tanglegram can be seen for *A*. *mirabilis* ‘V2221’ and *A*. *villosa* ‘L593.’

TreeMap analyses showed marginal (one node) to significant (29 nodes) support for 42 subtrees of the host phylogeny for the corresponding subtree in the parasite phylogeny, and showed marginal (one node) to significant (26 nodes) support for 46 subtrees of the parasite phylogeny for the corresponding subtree in the host phylogeny (S4 Fig, S4 Table).

**Fig 6:**
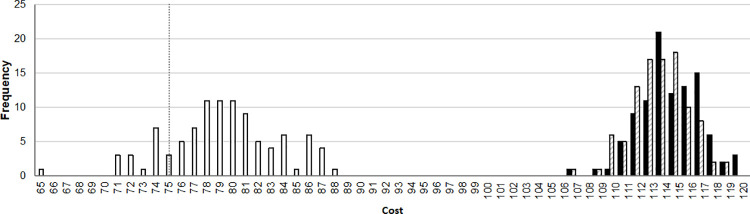
Distribution of costs of the reconstructions of the phylogenies. Null distribution of costs of the reconstructions of the independent phylogeny onto the dependent phylogeny using random tip mapping (RTM) and random parasite trees (RPT) for randomization are shown with slash pattern bars and black bars, respectively. Observed distribution of costs of the reconstruction of the bootstrapping-dependent phylogenies is shown with white bars. Observed cost (= 75) of the reconstruction of the consensus-independent phylogeny onto the consensus-dependent phylogeny is shown with a vertical dotted line.

Comparing the host and symbiont tanglegram using JANE (S5 Fig), 4901 optimal solutions were found. The 4901 solutions were compressed on 38 isomorphic solutions (solutions with the same cost and the same number of events for each event but the relative time, before or after a given node, is different). All of the solutions with an equal cost of 75. The optimal solutions presumed a maximum of 27 to 28 cospeciation events, from 3 to 5 loss events, from 35 to 36 duplication-with-host-switch events. Duplication and failure-to-diverge events were always 0. The most common isomorphic solution was repeated 903 times, and it was composed of 27 codivergence events, 0 duplication events, 36 duplication-with-host-switch events, 0 failure-to-diverge events, and 3 loss events. The overall cost of the historical reconstructions ranged from 65 to 88 (mean = 79.26, SD = 4.34) when phylogenetic uncertainty of host phylogeny was included (Fig 6). On the other hand, the total cost in the null distribution ranged from 107 to 119 (mean = 113.86, SD = 2.23) permuting the interactions between host and parasites (RTM), and from 106 to 119 (mean = 113.75, SD = 2.42) permuting the branches of the parasite tree (RPT, Fig 6). Permutation analyses showed that the observed total costs of the inferred historical reconstruction were significantly lower than those expected by chance (RTM, P< 0.001; RPT, P< 0.001). The three datasets, costs of ML bootstrap trees, and the two randomization sample costs, did not significantly deviate from normal distribution (ML bootstrap tree, W = 0.980, P = 0.122; RTM, W = 0.979, P = 0.104; RPT, W = 0.976, P = 0.068). The t-test significantly rejected the null hypotheses that the total costs of the inferred historical reconstruction of the pseudo-sampling bootstrap trees are equal to those expected by chance (RTM, P< 0.001; RPT, P< 0.001). These results show the concordance between the host and the symbiont phylogenies, despite having several host-switch events and loss events.

## Discussion

### Phylogenetic relationships within woody Myrsinoideae

Our nrITS phylogeny showed the monophyly of *Embelia* + *Myrsine* + *Heberdenia* + *Pleiomeris*, which was sister to other Myrsinoideae species in our sampling materials (Fig 1). This clade shared only few unique morphological characters among Myrsinoideae, such as the woody habit and consistent presence of functionally unisexual flowers. The position of *Embelia* was uncertain in different studies, including our nrITS phylogeny and two plastid markers phylogeny. The phylogenetic position of *Embelia* was either basal of the whole woody Myrsinoideae [20], sister to the woody Myrsinoideae except *Myrsine* and *Aegiceras* (the present two plastid markers phylogeny, S1 Fig; [21]), or sister to the *Myrsine*-*Heberdenia*-*Pleiomeris* clade (the present nrITS phylogeny, Fig 1). The phylogenetic incongruence may be explained by the different evolutionary history of the nuclear and plastid genes, such as sequence convergence, allopolyploidy, hybridization, introgression, or lineage sorting [52–54].

The genus *Rapanea* is one of the taxonomic ambiguities within Myrsinoideae, which has long been considered to be combined with *Myrsine*. *Rapanea* was separated from *Myrsine* based on the short and inconspicuous style and ligulate or capitate stigma (vs. obvious style and discoid or discoid-fimbriate stigma). Our phylogenetic works suggested that *Rapanea* should be included in *Myrsine*, as in the taxonomic revisions based on morphological studies completed by Pipoly and colleagues (e.g. [55–59]).

Both genera *Heberdenia* and *Pleiomeris* are monotypic and endemic to the Canary Islands. Our results are congruent with previous phylogenetic studies showing that the two genera are closely related, based on the morphological, nuclear external transcribed spacer (nrETS) and nrITS phylogenies [22,60,61]. These results also support Wit’s hypothesis that *Pleiomeris* is close to *Rapanea* (syn. *Myrsine*) [62], based on both reproductive and vegetative characters (i.e. unisexual flowers, sympetalous corolla, spurious prophylls covering the terminal bud and the young inflorescences, growth pattern, and flowering patterns). In fact, the monophyly of *Myrsine* (including *Rapanea*) has never been thoroughly examined by molecular phylogenetics, except of the study of Appelhans and colleagues [61], while the relationship of *Myrsine*-*Heberdenia*-*Pleiomeris* were poorly resolved. The phylogenetic relationship and taxonomy of *Myrsine*, *Heberdenia*, and *Pleiomeris* needs a more comprehensive study with more powerful molecular markers.

### Phylogenetic relationships within the *Ardisia* generic complex clade

Recent phylogenetic study using 78 plastid protein coding genes showed *Tapeinosperma* is sister to *Ardisia*, and the *Ardisia*-*Tapeinosperma* clade is sister to the *Parathesis*-*Elingamita* clade [36]. In our phylogeny based on two plastid IGS sequences, the results showed a similar relationship with Yan et al. [36], except for *Elingamita*, while *Labisia*, *Tapeinosperma* and *Ardisia* formed an unresolved tritomy (S1 Fig). In the phylogenetic works done by Julius and colleagues, *Labisia* was sister to the whole Asian *Ardisia* clade, and together the two were sister to the Neotropical species *A*. *opegrapha* Oerst., though the relationship was only moderately supported [20]. In other words, the genus *Tapeinosperma* is possibly nested in the *Ardisia* generic complex clade.

In the present study, we recognized at least four non-*Ardisia* genera were nested within the *Ardisia* generic complex clade, i.e. the *Badula*-*Oncostemum* clade, *Sadiria*, and *Hymenandra*. The results are compatible with previous phylogenetic studies, which revealed a total of 11 genera nested within the *Ardisia* generic complex clade [20,21]. The genus *Badula*, with 14 species, is endemic to the Mascarene Archipelago in the Western Indian Ocean. The genus *Oncostemum* is endemic to Madagascar and the Comoros with about 100 species, which is considered the most likely sister group to *Badula* on the basis of morphological characters and biogeographical distribution [63]. The two genera were distinguished by the fused stamens forming a tube surrounding the style in *Oncostemum* (versus distinct anthers in *Badula*), though the discriminating character is still controversial due to the discovery of intermediate forms [63,64]. Recent phylogenetic studies suggested that recognizing a monophyly of *Badula* will make *Oncostemum* paraphyletic [63,65], which is congruent with the morphological ambiguity. The discovery of the *Badula*-*Oncostemum* clade nested within the *Ardisia* generic complex clade is important in biogeographic study of the *Badula*-*Oncostemum* clade. The *Badula*-*Oncostemum* clade was hypothesized to have dispersed from Asia through Madagascar or the Mascarene islands rather than western Africa [65]. The *Badula*-*Oncostemum* clade was indicated to be sister to the *Ardisia* subg. *Pimelandra*, and *Pimelandra* is mainly distributed from southeast Asia to the Pacific islands (Figs 1 and 3; [20]). Unfortunately, no African *Ardisia* species have been included in the phylogenetic study of *Ardisia* and allies. It is still premature to infer the origin of the *Badula*-*Oncostemum* clade.

The genus *Hymenandra* was established by Spach in 1840, based on a section of *Ardisia* in Indo-Malesia established by De Candolle in 1834 [66,67]. It is characterized by united filaments (at least basally) and adnate to the corolla at its base, and anthers connate along the theca margin at least until anthesis [66,68]. The geographic distribution of *Hymenandra* has been extended to be amphi-Pacific tropical since Pipoly and Ricketson moved several Mesoamerican *Ardisia* species to *Hymenandra* in 1999 [69]. It is noteworthy that the filaments are connate only basally in the Indo-Malesian species, while they are completely fused in the neotropical species. Stone hypothesized *Hymenandra* is closely related to *Ardisia* subgenera *Pyrgus* (Lour.) Mez and *Crispardisia* based on morphological affinity [70]. Our molecular phylogeny suggested that *Hymenandra* is nested within the *Ardisia* generic complex (Figs 1 and 3), but the relationship between *Hymenandra* and *Ardisia* subgenera was unresolved.

The connate filaments and anthers are apparenly the only characters distinguishing *Hymenandra* from *Ardisia*. Pipoly and Ricketson found that the filaments in all *Ardisia* are actually connate basally to form a hyaline and inconspicuous tube, which is free from the corolla [18]. In our observation, the filaments indeed fuse and form a membranous and inconspicuous tube basally in many species across different subgenera, and the united part is shorter than or almost equal to that of the corolla. However, the stamen tube is often closely adnate to the corolla tube on fresh materials, making it difficult to determine whether the filament tube is completely free from the corolla. The fact that stamen tube is present in *Ardisia* may suggest that *Hymenandra* is a derived group within the *Ardisia* clade with extending stamen tube fusion level. In fact, stamen fusion, whether in filaments, anthers, or the whole stamens, has often been used to distinguish different genera in Myrsinoideae, such as *Amblyanthus* versus *Amblyanthopsis*, *Badula* versus *Oncostemum*, and *Ardisia* versus *Hymenandra* and *Conandrium* Mez. Our results and recent phylogenetic studies have revealed that this character evolved at least three times in the *Ardisia* generic complex clade (in *Amblyanthus*, *Oncostemum* and *Hymenandra*; Fig 3; [20,21]). The character apparently evolved repeatedly in Mysinoideae.

The *Sadiria* clade was also nested within the *Ardisia* generic complex clade in our analyses (Figs 1 and 3). The genus *Sadiria* was established by Mez in 1902 to accommodate those species with corolla lobes united to the middle and/or above [11], isolated from the genus *Pimelandra* A. DC. The remaining *Pimelandra* species with corolla lobes uniting only at the base were reduced to *Ardisia* subgenus *Pimelandra*. The genus *Sadiria* is mainly distributed from eastern Himalaya and Khasi Hills, extending to northern Myanmar and southwest China, and *Ardisia* subgenus *Pimelandra* is distributed in Peninsular Malaysia, Sumatra, Java, Borneo, Philippines, New Guinea, and Australia [6,8,9,11,71–76]. Hu and Deng claimed the genus *Sadiria* can be distinguished from *Ardisia* by the corolla and very short (with length equal to or shorter than the petioles), axillary, subfasciculate cymose, or subpaniculate inflorescence [74], but the description, except for the nearly fused corolla, completely falls into the circumscription of *Ardisia* subgenus *Pimelandra*. In other words, to our knowledge, *Sadiria* can only be distinguished from *Ardisia* by the proportion of corolla fusion. Nevertheless, no evidence showed the affinity of *Sadiria* and *Ardisia* subgenus *Pimelandra* in our study, and the relationship of *Sadiria* within the *Ardisia* generic complex clade is uncertain (Figs 1 and 3).

The specimen of *Ardisia* sp. ‘V4311’ was collected from southern Vietnam, with only fruits available. The specimen has multiple axillary fascicled infructescences with one to three fruits. The calyx has four to five lobes. These characters may resemble *Antistrophe caudata* King & Gamble. or *Ardisia fasciculiflora* C.M. Hu & J.E. Vidal (subgenus *Pimelandra*). Unfortunately, we don’t have flower material of *Ardisia* sp. ‘V4311’, which is the most important character to distinguish *Antistrophe* from *Ardisia*. The genus *Antistrophe* is distributed in India, Peninsular Malaysia, extending to Vietnam, which is distinct from *Ardisia* by the left-twisted floral buds [77]. If the specimen ‘V4311’ is *Antistrophe caudata*, *Antistrophe* is probably another genus that nested within the *Ardisia* generic complex clade. The answer will only be accessible if we can collect a flowering specimen of the material.

Further taxonomic revision dealing with the *Ardisia* generic complex is necessary. However, choosing whether to split *Ardisia* into several independent genera or to merge all nested genera into a broad sense *Ardisia* remain a difficult decision. Based on the phylogenetic studies so far, to incorporate all the nested genera into an enlarged *Ardisia* seems impractical, because with this treatment *Ardisia* would become a very large and heterogeneous genus. The acquisition of a broad and representative sample, covering the type species of *Ardisia* and the remaining genera of Myrsinoideae, is essential to the identification of well-supported clades that can be used as starting points for a more exhaustive study of systematics. In the present study, we tend not to make any formal nomenclatural change to the generic delimitation before a more comprehensive sampling and a robust phylogeny can be completed.

### Infrageneric classification of *Ardisia*

Based on our sampling materials, the grouping of the *Ardisia* species in our phylogeny was partially congruent with the present infrageneric classification (Figs 1–4). The subgenera *Crispardisia* was monophyletic, while the subgenera *Akosmos*, *Bladhia s*.*l*. and *Tinus* were paraphyletic or polyphyletic. The subgenera *Stylardisia* and *Pimelandra*, each consisting of only one species in our phylogeny, were distinct from other subgeneric clade, suggesting they are potentially natural groups. The subgeneric relationships are somewhat compatible with the results yielded by Julius and colleagues, although most of the subgeneric relationships remained unclear owing to insufficient resolution [20].

The subgenus *Bladhia s*.*l*. was not monophyletic and clearly showed two separated and highly supported clades (clade B and O, Figs 1 and 3). The two clades can be identified both by morphology and distribution range, corresponding to the two sections of *Bladhia s*.*l*., *Bladhia* and *Odontophylla* [78]. Here, we raised the section *Odontophylla* to a subgeneric level, and thus the circumscription of the subgenus *Bladhia* was modified and constricted. The description and species lists of the subgenera *Bladhia* (Thunb.) Mez emend. C.-J. Yang & J.-M. Hu (as *Bladhia s*.*str*. hereafter) and *Odontophylla* (Y.-P. Yang) C.-J. Yang & J.-M. Hu *stat*. *nov*. are provided below.

In our most broadly sampled subgenus, *Crispardisia*, at least two highly supported clades can be identified, clade C1 and C2 (Figs 2 and 4). Unfortunately, we failed to find any symapomorphic trait of either clade. *Ardisia villosa* Robx. is a species distributed in southern China, Taiwan, Indochina, Myanmar, and peninsular Malaysia. It is easily recognized by the villous or hirsute leaf and stem, marginal leaf nodules, and the calyx is almost equal to the corolla or fruit in length. Three specimens collected from different localities were included in our phylogenetic analyses, i.e. L593, Ku019, and Wang 2007237. Nevertheless, these specimens were all in different positions within clade C2. The result suggested that the taxonomic effort for this group is insufficient, and cryptic species may present. The *A*. *crenata* complex was firstly proposed and studied by Wang and Xia in 2012 [79], including four species and one variety (*A*. *crenata*, *A*. *hanceana*, *A*. *lindleyana* D. Dietr., *A*. *linangensis* C.M. Hu, *A*. *crenata* var. *bicolor*). They concluded *A*. *hanceana* and *A*. *lindleyana* are distinct from *A*. *crenata* based on both morphological and molecular evidence, and *A*. *linangensis* and *A*. *crenata* var. *bicolor* should be treated as synonym of *A*. *crenata*. Our results were mostly compatible with their study, but *A*. *hanceana* was nested within the *A*. *crenata* complex in our phylogeny. Furthermore, *A*. *polysticta* was also nested within the clade in our analyses, although the species can be distinguished from *A*. *crenata* by habit, leaf morphology, gland (not bacterial leaf nodule) color of leaf and flower, and fruit color.

The samples *A*. *humilis* Vahl ‘Hu4379’ and *A*. *porifera* ‘Zheng 822’ (accession: MF926213.1 and MF926089.1, direct submitted) were sister to each other and nested within clade T in our study (Fig 4). The taxonomic history of *A*. *humilis* is complicated, obstructing the identification of this species [7]. The name *A*. *humilis* was misapplied to *A*. *elliptica* Thunb. by Mez, which belongs to subgenus *Tinus* [11]. The name of the specimen ’Hu4379’ may be misapplied as the treatment of Mez, and the true identity of the ‘Hu4379’ is probably *A*. *elliptica* or a closely related species. *Ardisia porifera* belongs to the subgenus *Akosmos*. However, to distinguish the subgenus *Akosmos* from the genus *Tinus* is challenging, unless a good flowering material with well-developed inflorescence and clearly visible calyx lobes is available. Although we did not see the voucher specimen, we suspect the clade of *A*. *humilis* ‘Hu4379’ and *A*. *porifera* ‘Zheng 822’ may represent a group that is confusing and difficult to identify at the subgeneric level. Interestingly, clade T in the nrTIS phylogeny will be monophyetic if the clade of *A*. *humilis* ‘Hu4379’ and *A*. *porifera* ‘Zheng 822’ belongs to the subgenus *Tinus* (Fig 1).

The relationship among the subgenera is mostly unresolved or with weak support in both the present and previous studies (Figs 1–4; [20]). We found much conflicting information between major clades in the sequence alignments, and these sites are often highly correlated within clades. Morphologically, single diagnostic character is often used to define a subgenus, and unique characters can often be found in more than one subgenus. For instance, both lateral inflorescences and low ovule number occur in *Crispardisia* and *Akosmos*. Locellate anthers can be found both in *Tinus* and *Scherantha*. Both molecular and morphological characters suggest complicate relationships among *Ardisia* subgenera.

The recognition of smaller and monophyletic infrageneric groups within a diverse genus such as *Ardisia* is a critical step for future taxonomic revision. Having a more extensive sampling of *Ardisia* species in all subgenera, including species in the Neotropic, African, and Pacific Islands, and a robust phylogeny in future studies are necessary aspects of a robust and stable infrageneric classification. It is also important to have an understanding of the early evolution and biogeography of the genus *Ardisia*.

### Cophylogenetic analyses of the *Ardisia* subgenus *Crispardisia* and the associated *Burkholderia*

Both cophylogeneitc analyses and topology tests rejected the strict cospeciation evolutionary scenario in the bacterial leaf nodule symbiosis in *Ardisia*. The results agree with the preliminary study of Ku and Hu with 11 taxa pairs [19]. Although the phylogenies of the *Ardisia* host and their symbiotic *Burkholderia* are discordant, the tanglegram showed a tendency for associated plants and bacteria to occupy similar positions in phylogeny (Fig 5). The tanglegram with connected clades and species showed at least five or six corresponding subtrees, even with the phylogenetic uncertainties. Both the cophylogenetic analyses of TreeMap and Jane demonstrated a significant phylogenetic congruence between the *Ardisia* hosts and their symbionts, and both cospeciation and host-shift speciation also play important roles in evolution of the leaf nodule symbiotic relationship. However, the life history and ecological factors were often hypothesized to lead to a strict cospeciation in the *Ardisia*-*Burkholderia* association [80].

The *Ardisia*-*Burkholderia* symbiosis seems to provide an ideal condition for cospeciation occurring during the long-term evolution over the host shift speciation. Some well-known host-symbiont associations that are inferred to be strict or nearly strict cospeciation are often correlated with several features of life history and ecological factors (e.g. pocket gophers and their chewing lice [81]; *Brachycaudus* aphids and *Buchnera aphidicola* bacteria [82]). For instance, solitary and allopatric life history may reduce the likelihood of symbionts to shift their hosts [83]. Other factors such as the symbionts’ dispersal ability, the degree of specialization, the abundance of the host, and generation time of hosts and symbionts, may influence cospeciation probability. In the *Ardisia*-*Burkholderia* association, the symbionts are vertical transmitted, which guarantees that symbionts will acquire the next generation. In the *A*. *crenata* complex, all symbionts of the species complex are within a single lineage of bacterial phylogeny. The results showed one-to-one specificity of the *Ardisia*-*Burkholderia* association at species level. Furthermore, the *Burkholderia* symbionts are not able to be successfully cultured in agar medium and no free living *Burkholderia* species were found in the *Ardisia* symbiotic lineage [23], suggesting these symbionts have a limited ability to disperse. The intimate relationship not only restricts the transmitted pathway, but also implies that almost all potential hosts may already establish the symbiotic relationship.

Our results showed several lineages were not corresponding in the phylogenies of host and symbionts (Fig 5), suggesting host-shift speciation also play an important role in the evolution of the *Ardisia*-*Burkholderia* association. Unfortunately, to evaluate the importance of host-shift speciation in the evolution of the relationship is unlikely, because the species delimitation and phylogenetic resolution is not satisfied in our study. To show evidence and evaluate the frequency of a symbiont switching between closely to moderately related host species is difficult. In fact, strict congruence of phylogenies has rarely been found in the symbiotic relationship [80]. In the case of *Ardisia*-*Burkholderia* symbiosis, cospeciation is probably a nonadaptive process that occurs in the absence of selection. Cospeciation pattern could be achieved with repeated vicariance events followed by shared allopatric speciation, such as the Hawaiian silversword and its herbivores [83]. However, the chemical environment of the *Ardisia*-*Burkholderia* association has rarely been studied. Whether chemical coadaptation further reinforces host plant specialization and shapes the overall cospeciation pattern between *Ardisia* and *Burkholderia* is largely unknown.

To complete the process of host switching, the symbionts colonizing to a new host must drive the endemic symbiont extinct, or the symbiont must be primarily absent on the new host. The assumption of the former scenario is that coexistence of two bacterial species on a single host is not stable. Currently, no evidence shows that multiple *Burkholderia* species could coexist on a single host. The bacterial quantity is relatively low in the carpel, seed, and seedling [24], and the symbiont replacement could possibly be accomplished within these tissues. The alternative scenario is also plausible since the bacteria could be absent in the above tissues.

In the cases of bacterial leaf nodule symbiosis in Rubiacae, phylogenetic incongruence between hosts and symbionts and host-shift speciation have also been reported [26]. The life history and the ecological factors of the leaf nodule symbiosis in Rubiaceae is comparable to that in *Ardisia* [23,24]. The soil reservoir hypothesis seems unlikely to explain the mechanism of host switching, because both the symbiotic *Burkholderia* associating with *Ardisia* and Rubiaceae is unculturable and the reduced genome of the symbiotic *Burkholderia* may restrict them from living without a host [22,23,26,84]. In the Rubiaceae symbionts phylogeny, some soil isolates (*Burkholderia* sp. YI23 and *Burkholderia* sp. SJ98) and the stinkbug *Riptortus pedestris* symbiont (*Burkholderia* sp. RPE64) were nested in the clade [26]. Pinto-Carbó et al. hypothesized that the ancestor of symbiotic *Burkholderia* had a broad host range and may have had the ability to temporally associated with insects [26]. The insects, thus, could act as potential vector to transfer the symbiont to a new host, establishing a new symbiotic relationship (i.e. host-shift speciation). Although non-*Ardisia*-symbiotic *Burkholderia* sequence was not found in the *Ardisia* symbiont clade, the hypothesis may also be applied to the *Ardisia*-*Burkholderia* association. Another hypothesis to explain the host-switching process is that the symbiont could be transferred from the pollen donors, either from the same species or different species. Previous study has shown that symbionts can be detected in the stamens of *A*. *crenata* [19]. The bacteria could potentially be carried to different host species through pollinators, and then replace the native bacteria, establishing a new symbiotic relationship with the embryo in the seed. The process by which pollen and pollinators may serve as a vector for bacterial symbionts remains unclear.

### Taxonomic treatment

***Ardisia*** subgenus ***Bladhia*** (Thunb.) Mez emend. C.-J. Yang & J.-M. Hu

Type species: *Ardisia japonica* (Hornstedt) Blume

#### Description

Plants a small shrub with a creeping rhizome, pubescent with short round-capitate hairs on young stems, petioles, undersurface of leaves, and inflorescences. Hyposophylls present, oblanceolate, pubescent. Leaves subverticillate, subcoriaceous, serrulate, the lateral veins prominent, the reticulate veins distinct. Inflorescences umbellate or rarely umbellate-paniculate, axillary from hyposophylls or sometimes from leaves; calyx-lobes ovate or rarely long-ovate; ovules many in 2–3 whorls.

#### Note

The description is modified from Yang & Dwyer of *Ardisia* subg. *Bladhia* sect. *Bladhia* [78]. The reduced subgenus *Bladhia* now includes five species and one natural hybrid species in eastern China, Taiwan, the Ryukyus, Japan, and Korea, mainly distributed in temperate and subtropical regions.

### Species list

1. *Ardisia faberi* Hemsley
2. *Ardisia japonica* (Hornstedt) Blume
3. *Ardisia maclurei* Merr.
4. *Ardisia purpureovillosa* C.Y. Wu & C. Chen ex C.M. Hu
5. *Ardisia pusilla* A. DC.
6. *Ardisia* ×*walkeri* Y.P. Yang

Note: A natural hybrid of *A*. *japonica* and *A*. *pusilla*. See Kokubugata et al. [85].

***Ardisia*** subgenus ***Odontophylla*** (Y.P. Yang) C.-J. Yang and J.-M. Hu, *stat*. *nov*.

≡*Ardisia* subgenus *Bladhia* section *Odontophylla* Yang, in *Taiwania* 34(2): 232. 1989.

Type species: *Ardisia odontophylla* Wall. ex A. DC.

#### Description

Plants subshrubs or shrubs to 3 m high, with or without a creeping rhizome, pubescent with short round- or elongate-capitate or both kinds of hairs on young stem, petioles, undersurface of leaves, and inflorescences. Hypsophyll present, deltoid, linear or lanceolate. Leaves serrulate or fimbriate-serrulate. Inflorescences racemose, subumbellate, subumbellate-racemose, or paniculate, axillary from hyposophylls or leaves; calyx-lobes ovate or triangular-ovate, usually punctate; ovules 5–13 in a whorl.

#### Note

The description is modified from Yang & Dwyer of the *Ardisia* subg. *Bladhia* sect. *Odontophylla* [78]. The subgenus includes ca. 24 species and three varieties, which are distributed in Assam, northeastern India, southern and southwestern China, the Mainland Southeast Asia, Malay Peninsula, Java, and Borneo. The distributional range is mainly limited to tropical and subtropical regions. We listed several synonyms here, which are controversial in taxonomy and/or frequently used in regional flora.

### Species list

1. *Ardisia bambusetorum* King & Gamble
2. *Ardisia botryosa* Walker
3. *Ardisia bullata* G.H. Huang & G. Hao
4. *Ardisia cameronensis* Y.P. Yang

*syn*. *nov*. *Ardisia subverticillata* Julius & Utteridge

Note: Julius and Utteridge considered the name “*A*. *cameronensis* Y.P. Yang” to be invalid due to the lack of a Latin description in the original publication in 1989 [10], and they described a new species, *A*. *subverticillata* Julius & Utteridge, replacing it. However, Yang in fact had noticed the problem and validated the name *A*. *cameronensis* with Latin description later in *Bulletin of Taiwan Forest Research Institute* in the same year [86]. According to the *International Code of Nomenclature for algae*, *fungi*, *and plants (Shenzhen Code)* Article 33 [87], *A*. *cameronensis* should be a valid name, and *A*. *subverticillata* is thus a synonym of it. Although the epithet may not be properly reflecting the true distribution, *A*. *cameronensis* should be accepted and *A*. *subverticillata* should be abandoned.

5. *Ardisia curvistyla* Y.P. Yang
6. *Ardisia demissa* Miq. var. *demissa*
  *syn*. *Ardisia maingayi* (Clarke) King & Gamble
  *syn*. *Ardisia ordinata* Walker
7. *Ardisia demissa* Miq. var. *bambusetorum* (King & Gamble) C.M. Hu
8. *Ardisia dumicola* C.M. Hu & J.E. Vidal
9. *Ardisia fimbriata* Fletcher
10. *Ardisia foliosa* Furtado
11. *Ardisia gigantifolia* Stapf.
12. *Ardisia interjacens* C.M. Hu & J.E. Vidal
13. *Ardisia kteniophylla* A. DC.
14. *Ardisia metallica* N.E. Brown
15. *Ardisia nutantiflora* S.Z. Mao & C.M. Hu
16. *Ardisia odontophylla* Wall. ex A. DC.
17a. *Ardisia perpendicularis* Walker var. *perpendicularis*
17b. *Ardisia perpendicularis* Walker var. *balansana* (Yang) C.M. Hu
  *syn*. *Ardisia balansana* Y.P. Yang
18. *Ardisia pingbienensis* Y.P. Yang
  *syn*. *Ardisia longipedunculata* C.Y. Wu & C. Chen, non King & Gamble
19. *Ardisia theifolia* King & Gamble
20. *Ardisia ramondiaeformis* Pit.
21. *Ardisia replicata* Walker
  *syn*. *Ardisia pubivenula* Walker
22. *Ardisia scalarinervis* Walker
23a. *Ardisia silvestris* Pit. var. *silvestris*
  *syn*. *Ardisia dasyrhizomatica* C.Y. Wu & C. Chen
23b. *Ardisia silvestris* Pit. var. *appressa* C.M. Hu & J.E. Vidal
24. *Ardisia velutina* Pit.

## Conclusion

The present phylogenetic analyses of *Ardisia* and allies discovered current circumscription of the genus *Ardisia* is unsatisfactory and *Ardisia* is not monophyletic. The results showed that several *Ardisia* allied genera are nested within *Ardisia*, at least including *Badula*, *Oncostemum*, *Hymenandra*, and *Sadiria*. We identified at least three well-supported monophyletic lineages, corresponding to the infrageneric classification with taxonomic revision. Further taxa sampling and a robust phylogeny are necessary to understand the evolution of *Ardisia* and allies and to achieve a stable classification. Furthermore, this study has demonstrated that the *Ardisia* subg. *Crispardisia* phylogeny has significant topological congruence with the phylogeny of the *Burkholderia* symbionts, although strict cospeciation is not found. To date the divergent times of *Ardisia* and their symbionts would be crucial to further study in assessing whether the cases of topological congruence could be associated with cospeciation or host-shift speciation.

## Supporting information

S1 Fig Maximum likelihood (ML) phylogeny of *Ardisia* and allies based on plastid *psbA-trnH* and *rpl32-trnL* sequences. Numbers at branches are ML bootstrap support values.
(TIF)

S2 Fig Maximum likelihood (ML) phylogeny of the 16S rRNA sequence for *Burkholderia sensu lato*. Numbers at branches are ML bootstrap support values and Bayesian posterior probabilities. ML bootstrap support values are indicated only if at least 50, and the Bayesian posterior probabilities are indicated only if at least 0.7. Alternative branching between the two different analyses was shown as no support at the branch.
(TIF)

S3 Fig Maximum likelihood (ML) phylogeny of the 16S-23S rRNA sequence for the *Ardisia* symbionts. Numbers at branches are ML bootstrap support values. ML bootstrap support values are indicated only if at least 50.
(TIF)

S4 Fig Tanglegram for leaf-nodulated *Ardisia* and their symbiotic bacteria. Results from distance-based analysis using TreeMap are shown. Symbiont species (yellow, right) are connected to their host (blue, left) by gray lines. Maximum likelihood trees are based on the nrITS and plastid sequences (*psbA-trnH* and *rpl32-trnL*) for the host (left) and the 16S-23S rRNA of their symbiotic bacteria (right). Outgroups were removed here. Significant nodes are shown in red and the degree of significance is shown by the intensity of color.
(TIF)

S5 Fig Cophylogeny reconstruction of the history of host-parasite interaction between leaf-nodulated *Ardisia* and their symbiotic *Burkholderia*. One of the most common isomorphic solutions using the event-based method in Jane. The *Ardisia* phylogeny is shown in black and the symbionts’ phylogeny is shown in blue. Cospeciation event is marked by an open and colored circle, duplication event is marked by a solid and colored circle, and duplication-with-host-switch event is marked by a duplication event with an arrow following the trajectory of the switching species. A yellow node indicates that there is another location of equal cost, and a red node means that all other locations to which it may be mapped are of higher cost.
(TIF)

S1 Table Voucher details and GenBank accession numbers of *Ardisia*, allies, and outgroups. (DOCX)

S2 Table Primers used for PCR and sequencing in this study. (DOCX)

S3 Table Detailed information of the *Burkholderia s*.*l*. sequences used in this study. (DOCX)

S4 Table Statistic values of comparing congruence within subtrees in TreeMap analysis. (DOCX)
