# Supplementary figures and images for: Molecular phylogeny of Asian Ardisia (Myrsinoideae, Primulaceae) and their leaf-nodulated endosymbionts, Burkholderia s.l. (Burkholderiaceae)
Source: PLoS One. 2022 Jan 19;17(1):e0261188. doi: 10.1371/journal.pone.0261188 (PMC8769342; doi:10.1371/journal.pone.0261188)

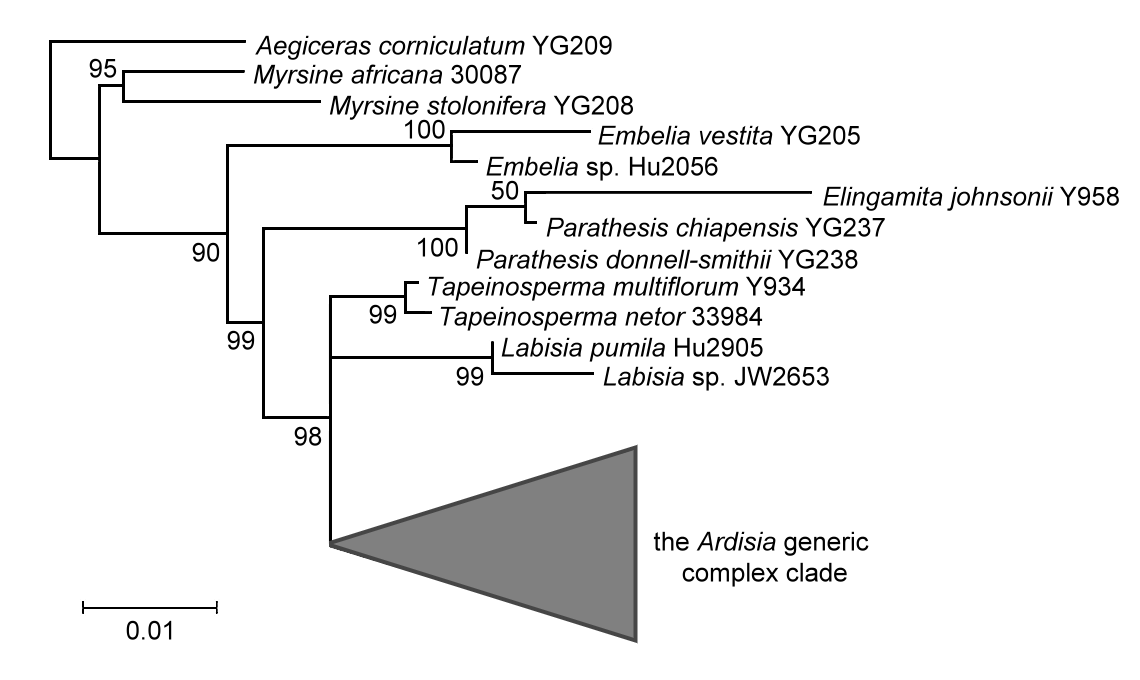

Supplement: S1 Fig — Numbers at branches are ML bootstrap support values. (TIF) [file pone.0261188.s001.tif]

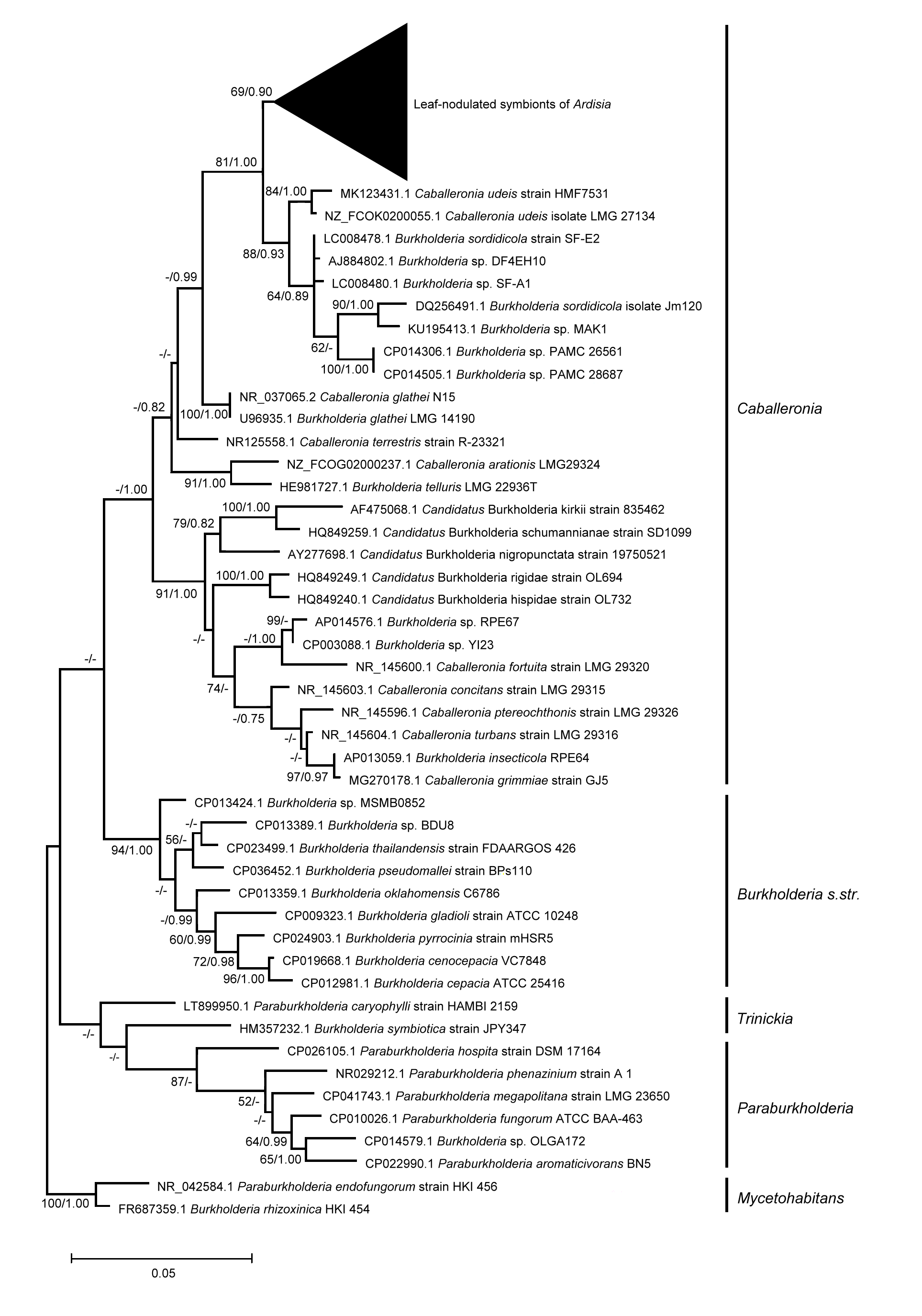

Supplement: S2 Fig — Numbers at branches are ML bootstrap support values and Bayesian posterior probabilities. ML bootstrap support values are indicated only if at least 50, and the Bayesian posterior probabilities are indicated only if at least 0.7. Alternative branching between the two different analyses was shown as no support at the branch. (TIF) [file pone.0261188.s002.tif]

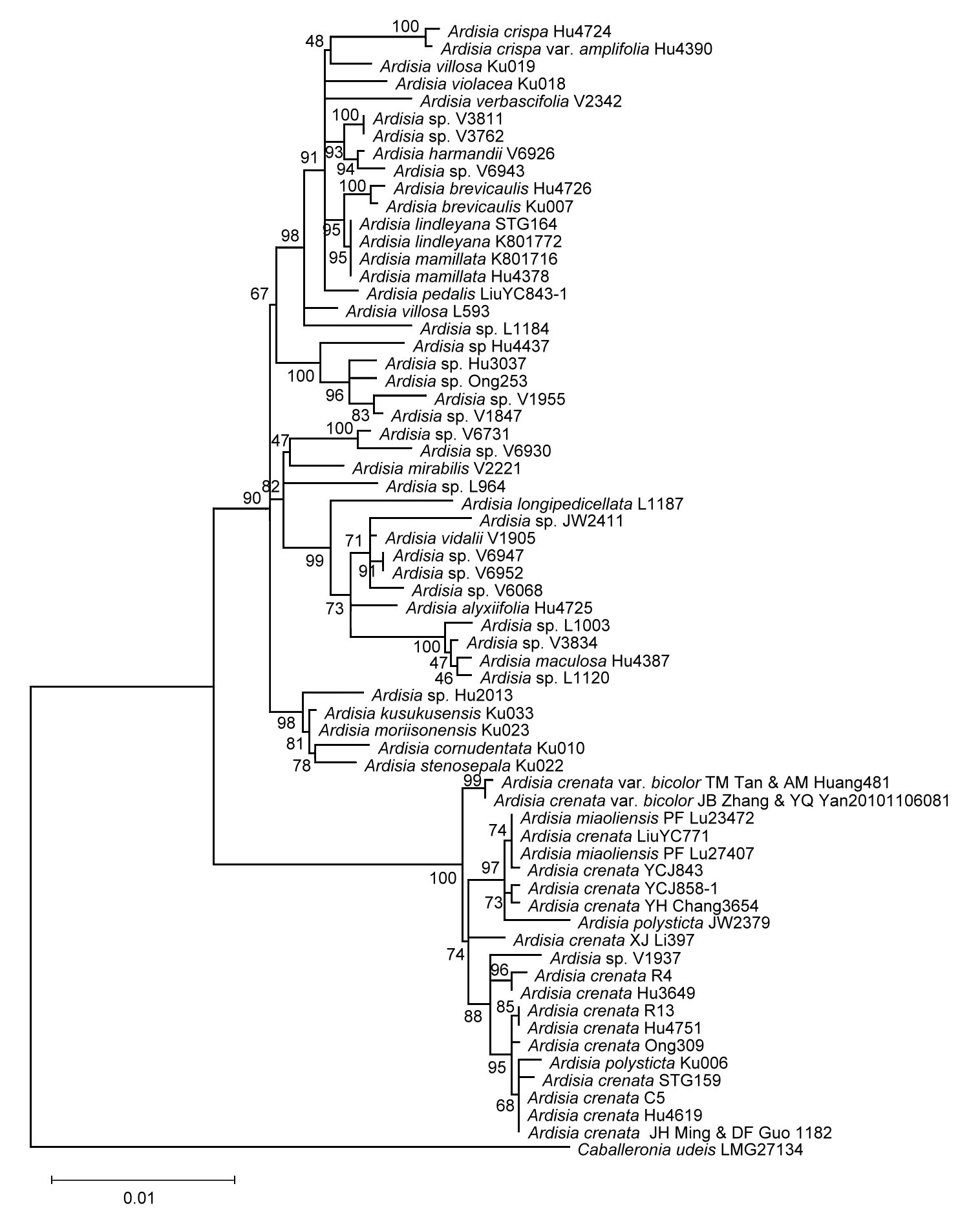

Supplement: S3 Fig — Numbers at branches are ML bootstrap support values. ML bootstrap support values are indicated only if at least 50. (TIF) [file pone.0261188.s003.tif]

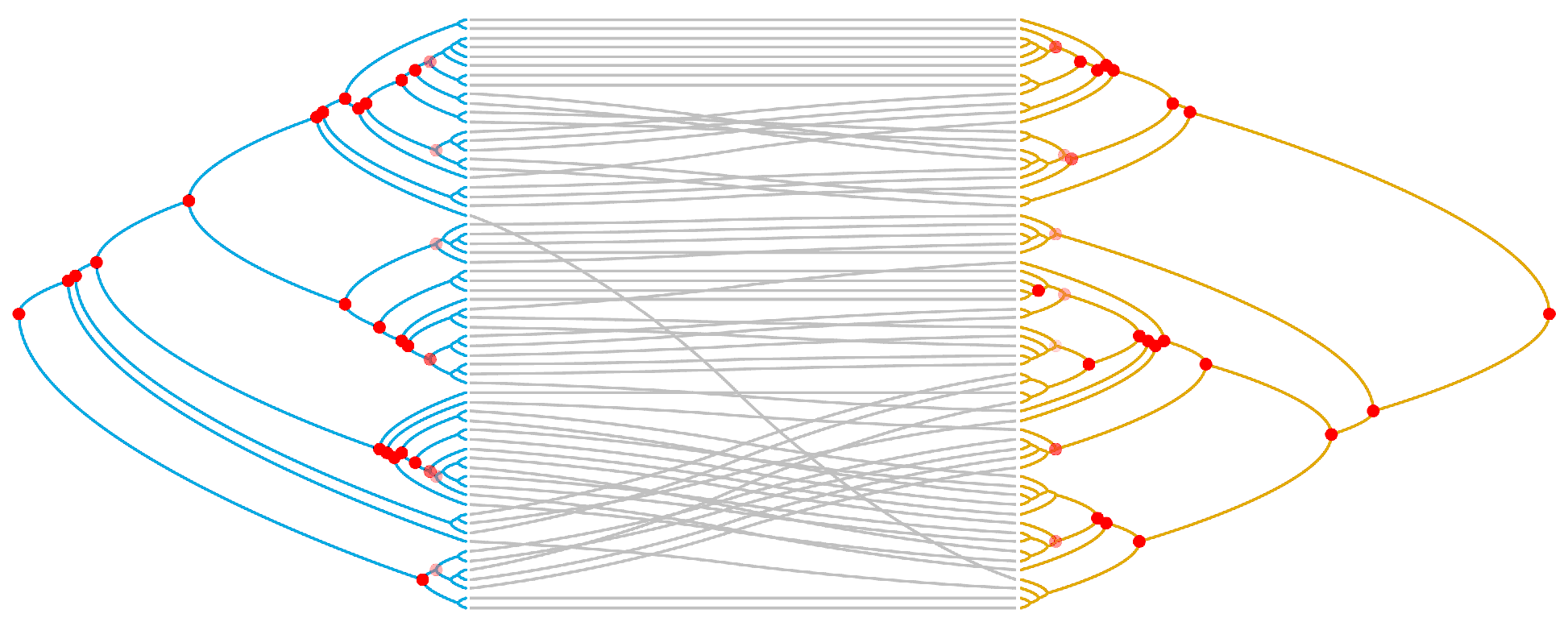

Supplement: S4 Fig — Results from distance-based analysis using TreeMap are shown. Symbiont species (yellow, right) are connected to their host (blue, left) by gray lines. Maximum likelihood trees are based on the nrITS and plastid sequences (psbA-trnH and rpl32-trnL) for the host (left) and the 16S-23S rRNA of their symbiotic bacteria (right). Outgroups were removed here. Significant nodes are shown in red and the degree of significance is shown by the intensity of color. (TIF) [file pone.0261188.s004.tif]

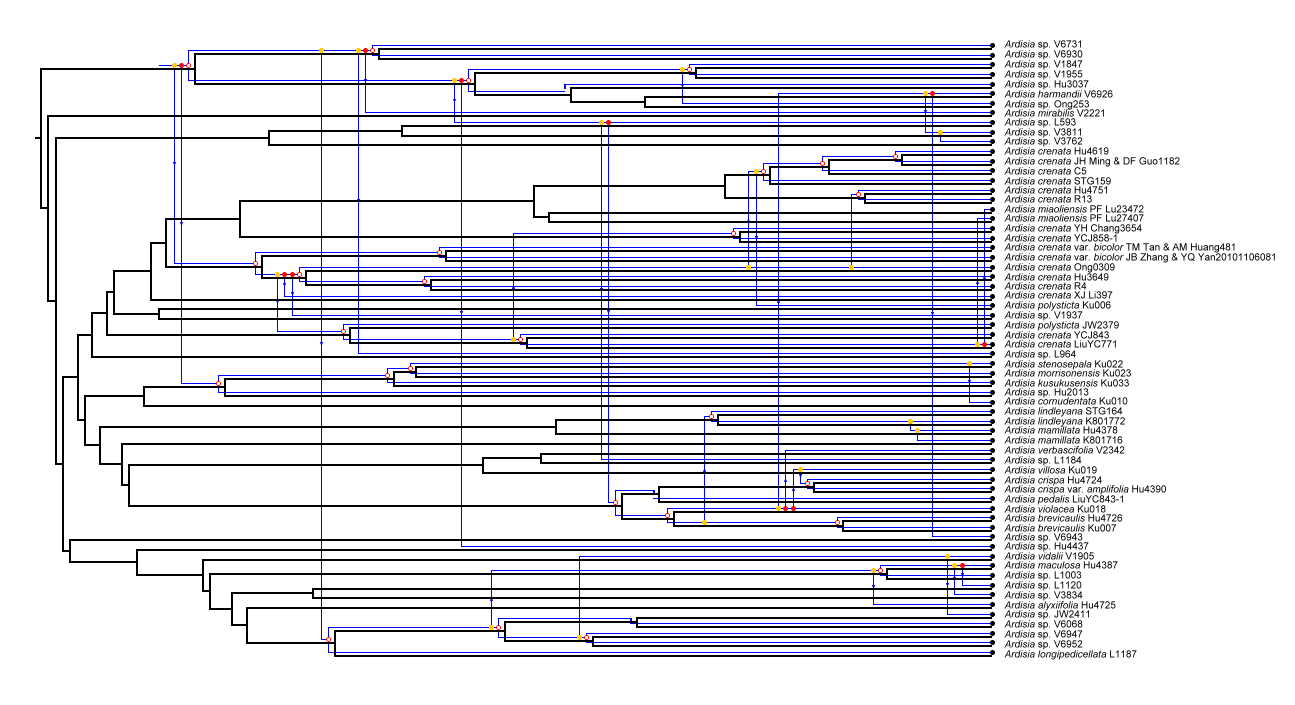

Supplement: S5 Fig — One of the most common isomorphic solutions using the event-based method in Jane. The Ardisia phylogeny is shown in black and the symbionts’ phylogeny is shown in blue. Cospeciation event is marked by an open and colored circle, duplication event is marked by a solid and colored circle, and duplication-with-host-switch event is marked by a duplication event with an arrow following the trajectory of the switching species. A yellow node indicates that there is another location of equal cost, and a red node means that all other locations to which it may be mapped are of higher cost. (TIF) [file pone.0261188.s005.tif]
