# Supplementary material for: Molecular phylogeny of Asian Ardisia (Myrsinoideae, Primulaceae) and their leaf-nodulated endosymbionts, Burkholderia s.l. (Burkholderiaceae)
Source: PLoS One. 2022 Jan 19;17(1):e0261188. doi: 10.1371/journal.pone.0261188 (PMC8769342; doi:10.1371/journal.pone.0261188)
Supplement: S1 Table — (DOCX) [file pone.0261188.s006.docx]

**S1 Table. Voucher details and GenBank accession numbers of *Ardisia*, allies, and outgroups.**

| Taxon | nrITS | *psbA-trnH* | *rpl32-trnL* | 16S-23S *rRNA* of symbiont | Voucher (Herbarium code, if available) | Synonyms or misapplied name |
| --- | --- | --- | --- | --- | --- | --- |
| ***Ardisia alyxiifolia* Tsiang ex C.Chen** | MW414707 | MW456338 | MW456436 | MW467993 | Hu4725 (TAI) |  |
| *Ardisia argenticaulis* Yuen P.Yang | MF926183 | MF926054 |  |  | Wang200848 |  |
| *Ardisia attenuata* Wall. & A.DC. | MF926184 | MF926055 |  |  | Xia et al.2670 |  |
| *Ardisia balansana* Yuen P.Yang | MF926209 | MF926106 |  |  | Wang200621 |  |
| *Ardisia baotingensis* C.M.Hu | MF926185 | MF926056 |  |  | Wang200791 |  |
| ***Ardisia brevicaulis* Diels** | MW414708 | MW456339 | MW456420 | MW467994 | Hu4726 (TAI) |  |
| ***Ardisia brevicaulis* Diels** | JQ684765 | JQ684786 | MW456430 | JQ684813 | Ku007 (TAI) |  |
| *Ardisia brunnescens* Walker | MW414690 | MW456335 | MW456438 |  | Hu4376 (TAI) |  |
| ***Ardisia caudata* Hemsl.** | MF926188 | MF926059 |  |  | Wang2007194 |  |
| *Ardisia chevalieri* Pit. | MW414744 | MW456342 | MW456419 |  | V2345 (TAI) |  |
| *Ardisia chinensis* Benth. | MW414696 | MW456336 | MW456437 |  | Hu4384 (TAI) |  |
| *Ardisia collinsiae* Fletcher | MW414687 | MW456341 | MW456439 |  | Hu3034 (TAI) |  |
| ***Ardisia conspersa* Walker** | MF926190 | MF926061 |  |  | Wang2007260 |  |
| ***Ardisia cornudentata* Mez** | JQ684760 | JQ684784 | MW456432 | JQ684807 | Ku010 (TAI) |  |
| ***Ardisia corymbifera* Mez** | MF926191 | MF926062 |  |  | Wang200623 |  |
| ***Ardisia crenata* Sims** | MW414681 | MW456333 | MW456448 | MW467990 | C5 (MAK) |  |
| ***Ardisia crenata* Sims** | MW414689 | MW456334 | MW456447 | MW467991 | Hu3649 (TAI) |  |
| ***Ardisia crenata* Sims** | MW414704 | MW456337 | MW456446 | MW467992 | Hu4619 (TAI) |  |
| ***Ardisia crenata* Sims** | MW414709 | MW456340 | MW456445 | MW467995 | Hu4751 (TAI) |  |
| ***Ardisia crenata* Sims** | MW414723 | MW456343 | MW456444 | MW467996 | LiuYC771 (TAI) |  |
| ***Ardisia crenata* Sims** | MW414725 | MW456344 | MW456443 | MW467997 | Ong0309 (TAI) |  |
| ***Ardisia crenata* Sims** | MW414727 | MW456347 | MW456441 | MW467999 | R13 (MAK) |  |
| ***Ardisia crenata* Sims** | MW414728 | MW456346 | MW456442 | MW467998 | R4 (MAK) |  |
| ***Ardisia crenata* Sims** | MW414729 | MW456345 | MW456440 | MW468000 | STG159 (TAI) |  |
| ***Ardisia crenata* Sims** | MW414761 | MW456348 | MW456451 | MW468044 | YCJ843 (TAI) |  |
| ***Ardisia crenata* Sims** | MW414763 | MW456349 | MW456450 | MW468045 | YCJ858-1 (TAI) |  |
| ***Ardisia crenata* Sims** | MW414765 | MW456350 | MW456449 |  | YCJ860 (TAI) |  |
| ***Ardisia crenata* Sims** | MW414731 | MW456351 | MW456457 | MW468039 | Y.-H. Chang3654 (TAIF) |  |
| ***Ardisia crenata* Sims** | MW414735 | MW456354 | MW456454 | MW468042 | X.-J. Li397 (TAIF) |  |
| ***Ardisia crenata* Sims** | MW414737 | MW456355 | MW456452 | MW468043 | J.-H. Ming & D.-F. Guo1182 (TAIF) |  |
| ***Ardisia crenata* var. *bicolor* (Walker) C.Y.Wu & C.Chen** | MW414732 | MW456352 | MW456458 | MW468040 | T.-M. Tan & A.-M. Huang481 (TAIF) |  |
| ***Ardisia crenata* var. *bicolor* (Walker) C.Y.Wu & C.Chen** | MW414734 | MW456353 | MW456455 | MW468041 | J.-B. Zhang & Y.-Q. Yan20101106081 (TAIF) |  |
| ***Ardisia crispa* (Thunb.) A.DC.** | MW414706 | MW456362 | MW456462 | MW468038 | Hu4724 (TAI) |  |
| ***Ardisia crispa* (Thunb.) A.DC.** | MW414764 | MW456365 | MW456459 | MW468046 | YCJ859 (TAI) |  |
| ***Ardisia crispa* var. *amplifolia* Walker** | MW414701 | MW456360 | MW456464 | MW468037 | Hu4390 (TAI) |  |
| *Ardisia cymosa* Blume | JQ684773 | JQ684803 | MW456421 |  | Ku014 (TAI) |  |
| *Ardisia densilepidotula* Merr. | MF926194 | MF926066 |  |  | Wang200787 |  |
| *Ardisia depressa* C.B.Clarke | JN645199 | MF926067 |  |  | Wang2007169 |  |
| *Ardisia elliptica* Thunb. | JQ684776 | JQ684798 | MW456422 |  | Ku030 (TAI) |  |
| ***Ardisia ensifolia* Walker** | MF926195 | MF926069 |  |  | Wang2007210 |  |
| *Ardisia faberi* Hemsl. | MW414702 | MW456361 | MW456463 |  | Hu4391 (TAI) |  |
| ^a^***Ardisia filiformis* Walker** | MF926196 | MF926070 |  |  | Wang2007161 |  |
| *Ardisia fordii* Hemsl. | MW414697 | MW456359 | MW456465 |  | Hu4385 (TAI) |  |
| *Ardisia gigantifolia* Stapf | MW414691 | MW456356 | MW456468 |  | Hu4377 (TAI) |  |
| *Ardisia gigantifolia* Stapf | MW414751 | MW456363 | MW456461 |  | V6371 (TAI) |  |
| *Ardisia gigantifolia* Stapf | MF926198 | MF926072 |  |  | Wang2007139 |  |
| ***Ardisia hanceana* Mez** | MF926199 | MF926073 |  |  | Wang2007185 |  |
| ***Ardisia harmandii* Pierre ex Pit.** | MW414755 | MW456364 | MW456460 | MW468047 | V6926 (TAI) |  |
| *Ardisia humilis* Vahl | MW414693 | MW456357 | MW456467 |  | Hu4379 (TAI) |  |
| *Ardisia hypargyrea* C.Y. Wu & C. Chen | MF926232 | MF926108 |  |  | Wang2007192 | *Ardisia quinquegona* var. *salicifolia* (E. Walker) C.M. Hu & J.E. Vidal |
| *Ardisia japonica* Blume | MW414694 | MW456358 | MW456466 |  | Hu4381 (TAI) |  |
| *Ardisia japonica* Blume | JQ684778 | JQ684801 | MW456426 |  | Ku008 (TAI) |  |
| ***Ardisia kachinensis* Mez** | MF926201 | MF926076 |  |  | Gao et al.2726 |  |
| ***Ardisia kusukusensis* Hayata** | JQ684761 | JQ684785 | MW456431 | JQ684809 | Ku033 (TAI) |  |
| ***Ardisia lindleyana* D.Dietr.** | MW414715 | MW456369 | MW456473 | MW468004 | K801772 (KBCC) |  |
| ***Ardisia lindleyana* D.Dietr.** | MW414730 | MW456371 | MW456470 | MW468006 | STG164 (TAI) |  |
| ***Ardisia longipedicellata* H.R. Fletcher** | MW414720 | MW456370 | MW456472 | MW468005 | L1187 (TAI) |  |
| *Ardisia maclurei* Merr. | JQ684780 | JQ684800 | MW456429 |  | Ku015 (TAI) |  |
| ***Ardisia maculosa* Mez** | MW414699 | MW456367 | MW456476 | MW468002 | Hu4387 (TAI) |  |
| ***Ardisia mamillata* Hance** | MW414692 | MW456366 | MW456477 | MW468001 | Hu4378 (TAI) |  |
| ***Ardisia mamillata* Hance** | MW414714 | MW456368 | MW456474 | MW468003 | K801716 (KBCC) |  |
| ***Ardisia miaoliensis* S.Y. Lu** | MW414733 | MW456378 | MW456456 | MW468010 | P.-F. Lu23472 (TAIF) |  |
| ***Ardisia miaoliensis* S.Y. Lu** | MW414736 | MW456379 | MW456453 | MW468011 | P.-F. Lu27407 (TAIF) |  |
| ***Ardisia mirabilis* Pit.** | MW414742 | MW456372 | MW456469 | MW468007 | V2221 (TAI) |  |
| ***Ardisia morrisonensis* Hayata** | JQ684759 | JQ684783 | MW456433 | JQ684808 | Ku023 (TAI) | *Ardisia cornudentata* subsp. *morrisonensis* (Hayata) Yuen P.Yang |
| *Ardisia obtusa* Mez | MF926205 | MF926082 |  |  | Wang2007302 |  |
| ***Ardisia omissa* C.M.Hu** | MF926206 | MF926083 |  |  | Wang2007117 |  |
| *Ardisia ordinata* Walker | MF926207 | MF926084 |  |  | Chen2007294 |  |
| *Ardisia pachysandra* (Wall.) Mez | MW414686 | MW456376 | MW456478 |  | Hu3033 (TAI) |  |
| ***Ardisia pedalis* Walker** | MW414724 | MW456375 | MW456471 | MW468009 | LiuYC843-1 (TAI) |  |
| *Ardisia perpendicularis* Walker | MF926208 | MF926085 |  |  | Wang200711 |  |
| *Ardisia pingbienensis* Yuen P.Yang | MF926210 | MF926086 |  |  | Wang2007225 |  |
| *Ardisia polycephala* Wall. & A.DC. | MF926211 | MF926087 |  |  | Xia et al.384 |  |
| ***Ardisia polysticta* Miq.** | JQ684771 | JQ684795 | MW456434 | JQ684819 | Ku006 (TAI) |  |
| ***Ardisia polysticta* Miq.** | MW414710 | MW456377 | MW456475 | MW468008 | JW2379 (TAI) |  |
| *Ardisia porifera* Walker | MF926213 | MF926089 |  |  | Zheng822 |  |
| ***Ardisia primulifolia* Gardner & Champ.** | MF926214 | MF926090 |  |  | Wang2007195 |  |
| ***Ardisia pseudocrispa* Pit.** | MF926215 | MF926091 |  |  | Wang2007228 |  |
| *Ardisia pubivenula* Walker | MF926217 | MF926092 |  |  | Wang200795 |  |
| *Ardisia purpureovillosa* C.Y.Wu & C.Chen ex C.M.Hu | MW414698 | MW456374 | MW456485 |  | Hu4386 (TAI) |  |
| *Ardisia pusilla* A.DC. | MW414695 | MW456373 | MW456486 |  | Hu4383 (TAI) |  |
| *Ardisia pusilla* A.DC. | JQ684779 | JQ684799 | MW456423 |  | Ku017 (TAI) |  |
| *Ardisia quinquegona* Blume | MW414700 | MW456380 | MW456484 |  | Hu4389 (TAI) |  |
| *Ardisia quinquegona* Blume | JQ684774 | JQ684804 | MW456424 |  | Ku002 (TAI) |  |
| *Ardisia sanguinolenta* Blume | MW414684 | MW456386 | MW456487 |  | Hu2904 (TAI) |  |
| *Ardisia scalarinervis* Walker | MF926221 | MF926096 |  |  | Wang2007233 |  |
| *Ardisia sieboldii* Miq. | JQ684775 | JQ684802 | MW456425 |  | Ku027 (TAI) |  |
| *Ardisia silvestris* var. *appressa* C.M.Hu & J.E.Vidal | MF926222 | MF926109 |  |  | Wang200770 |  |
| ***Ardisia sinoaustralis* C.Chen** | MF926181 | MF926052 |  |  | Wang2007297 | *Ardisia affinis* Hemsley (1889), non A.DC. (1844) |
| *Ardisia solanacea* Roxb. | MF926223 | MF926097 |  |  | Wang2007213 |  |
| *Ardisia squamulosa* C.Presl | MW414766 | MW456390 | MW456479 |  | YCJ879 (TAI) |  |
| ***Ardisia stenosepala* Hayata** | JQ684758 | JQ684782 | MW456428 | JQ684806 | Ku022 (TAI) | *Ardisia cornudentata* var. *stenosepala* (Hayata) Yuen P.Yang |
| *Ardisia tenera* Mez | MF926225 | MF926099 |  |  | Wang2007239 |  |
| *Ardisia theifolia* King & Gamble | MW414712 | MW456383 | MW456483 |  | JW2438 (TAI) |  |
| *Ardisia thyrsiflora* D.Don | MF926226 | MF926100 |  |  | Gao et al.2808 |  |
| ***Ardisia verbascifolia* Mez** | MW414743 | MW456389 | MW456480 | MW468027 | V2342 (TAI) |  |
| ***Ardisia vidalii* C.M.Hu** | MW414739 | MW456388 | MW456481 | MW468024 | V1905 (TAI) |  |
| ***Ardisia villosa* Roxb.** | MW414721 | MW456384 | MW456482 | MW468016 | L593 (TAI) |  |
| ***Ardisia villosa* Roxb.** | JQ684767 | JQ684791 | MW456435 | JQ684815 | Ku019 (TAI) |  |
| ***Ardisia villosa* var. *oblanceolata* Walker** | MF926229 | MF926110 |  |  | Wang2007237 |  |
| ***Ardisia violacea* (T.Suzuki) W.Z.Fang & K.Yao** | JQ684766 | JQ684790 | MW456427 | JQ684812 | Ku018 (TAI) |  |
| ^a^*Ardisia waitakii* C.M.Hu | MF926230 | MF926103 |  |  | Wang2007223 |  |
| ***Ardisia* sp.** | MF926229 | MF926110 | MW456513 | MW468012 | Hu2013 (TAI) |  |
| ***Ardisia* sp.** | JQ684766 | JQ684790 | MW456427 | JQ684812 | Hu3037 (TAI) |  |
| ***Ardisia* sp.** | MF926230 | MF926103 | MW456511 | MW468014 | Hu4437 (TAI) |  |
| ***Ardisia* sp.** | MW414682 | MW456385 | MW456513 | MW468012 | JW2411 (TAI) |  |
| ***Ardisia* sp.** | MW414688 | MW456387 | MW456512 | MW468013 | L964 (TAI) |  |
| ***Ardisia* sp.** | MW414716 | MW456392 | MW456509 | MW468018 | L1002 (TAI) |  |
| ***Ardisia* sp.** | MW414717 | MW456393 | MW456508 | MW468019 | L1003 (TAI) |  |
| ***Ardisia* sp.** | MW414718 | MW456394 | MW456507 | MW468020 | L1120 (TAI) |  |
| ***Ardisia* sp.** | MW414719 | MW456395 | MW456506 | MW468021 | L1184 (TAI) |  |
| ***Ardisia* sp.** | MW414726 | MW456396 | MW456505 | MW468022 | Ong253 (TAI) |  |
| ***Ardisia* sp.** | MW414738 | MW456397 | MW456504 | MW468023 | V1847 (TAI) |  |
| ***Ardisia* sp.** | MW414740 | MW456398 | MW456503 | MW468025 | V1937 (TAI) |  |
| ***Ardisia* sp.** | MW414741 | MW456400 | MW456502 | MW468026 | V1955 (TAI) |  |
| ***Ardisia* sp.** | MW414745 | MW456401 | MW456501 |  | V3589 (TAI) |  |
| ***Ardisia* sp.** | MW414746 | MW456402 | MW456500 | MW468028 | V3762 (TAI) |  |
| ***Ardisia* sp.** | MW414747 | MW456403 | MW456499 | MW468029 | V3811 (TAI) |  |
| ***Ardisia* sp.** | MW414748 | MW456404 | MW456498 | MW468030 | V3834 (TAI) |  |
| *Ardisia* sp. | MW414749 | MW456405 | MW456497 |  | V4311 (TAI) |  |
| ***Ardisia* sp.** | MW414750 | MW456406 | MW456496 | MW468031 | V6068 (TAI) |  |
| ***Ardisia* sp.** | MW414752 | MW456407 | MW456495 |  | V6372 (TAI) |  |
| *Ardisia* sp. | MW414753 | MW456408 | MW456494 |  | V6730 (TAI) |  |
| ***Ardisia* sp.** | MW414754 | MW456409 | MW456493 | MW468032 | V6731 (TAI) |  |
| ***Ardisia* sp.** | MW414756 | MW456410 | MW456492 | MW468033 | V6930 (TAI) |  |
| ***Ardisia* sp.** | MW414757 | MW456411 | MW456491 | MW468034 | V6943 (TAI) |  |
| ***Ardisia* sp.** | MW414758 | MW456412 | MW456490 | MW468035 | V6947 (TAI) |  |
| ***Ardisia* sp.** | MW414759 | MW456413 | MW456489 | MW468036 | V6952 (TAI) |  |
| *Ardisia* sp. | MW414762 | MW456399 | MW456488 |  | YCJ851 (TAI) |  |
| *Badula crassa* A.DC. | HE590619 | HF548965 |  |  | R. Bone22 |  |
| *Badula sieberi* A.DC. | HE590655 | HF549000 |  |  | R. Bone12 |  |
| *Hymenandra wallichii* A.DC. | MF926193 | MF926065 |  |  | Wang2007212 | ^b^*Ardisia dasyrhizomatica* C.Y.Wu & C.Chen |
| *Oncostemum* sp. | HF548947 | HF549007 |  |  | TDNA:503 |  |
| *Sadiria aberrans* (Walker) C.M.Hu & Y.F.Deng | JN645198 | MF926051 |  |  | Xia et al.381 | ^b^*Ardisia aberrans* (Walker) C.Y. Wu & C. Chen |
| *Sadiria* sp. | LT964874 |  |  |  | L ZW-2017 |  |
| Outgroups |  |  |  |  |  |  |
| *Aegiceras corniculatum* (L.) Blanco | MH243956 |  |  |  | isolate b2m23 |  |
| *Embelia ribes* Burm.f. | MG877757 |  |  |  | GBOWS1393 |  |
| *Embelia* sp. | MW414683 | MW456416 | MW456519 |  | Hu2056 (TAI) |  |
| *Heberdenia excelsa* Banks ex Roem. & Schult. | KJ189030 |  |  |  |  |  |
| *Labisia pumila* Benth. & Hook. f. | MW414685 | MW456417 | MW456517 |  | Hu2905 (TAI) |  |
| *Labisia* sp. | MW414713 | MW456415 | MW456515 |  | JW2653 (TAI) |  |
| *Monoporus bipinnatus* Mez | HF548941 |  |  |  | TDNA:846 |  |
| *Myrsine faberi* (Mez) Pipoly & C.Chen | MG877849 |  |  |  | Hao269 | *Rapanea faberi* Mez |
| *Myrsine kwangsiensis* (E.Walker) Pipoly & C.Chen | MW414705 | MW456414 | MW456516 |  | Hu4708 | *Rapanea kwangsiensis* Walker |
| *Myrsine seguinii* H.Lév. | MW414760 | MW456418 | MW456514 |  | YCJ825 (TAI) |  |
| *Myrsine seguinii* H.Lév. | MG877853 |  |  |  | GBOWS375 |  |
| *Myrsine semiserrata* Wall. | MG877850 |  |  |  | GBOWS263 |  |
| *Pleiomeris canariensis* A.DC. | KJ189027 |  |  |  |  |  |
| *Stylogyne* sp. | AF164019 |  |  |  | Staahl & Knudsen1363 |  |

Herbarium vouchers collected by authors were deposited at KBCC, TAI, TAIF and MAK. Species with leaf nodules were shown in boldface.

^a^Presumed misidentification. ^b^Misapplied name suggested by other authors.
