# Supplementary material for: Molecular phylogeny of Asian Ardisia (Myrsinoideae, Primulaceae) and their leaf-nodulated endosymbionts, Burkholderia s.l. (Burkholderiaceae)
Source: PLoS One. 2022 Jan 19;17(1):e0261188. doi: 10.1371/journal.pone.0261188 (PMC8769342; doi:10.1371/journal.pone.0261188)
Supplement: S2 Table — (DOCX) [file pone.0261188.s007.docx]

**S2 Table. Primers used for PCR and sequencing in this study.**

| **Primers** | **Sequence (5' to 3')** | **Region/position** | **Notes** | **Reference** |
| --- | --- | --- | --- | --- |
| Myrsinoidea, Primulaceae | | | | |
| ITS6 | AAC TCA GCG GGT AAT CCC G | Nuclear ITS1, 5,8S, and ITS2 |  | Ku & Hu, 2014 |
| ITS7 | GTC GTA ACA AGG TTT CCG TAG |  |  |  |
|  |  |  |  |  |
| psbA-3F | GTT ATG CAT GAA CGT AAT GCT C | Plastid *psbA*-*trnH* |  | Sang et al., 1997 |
| trnH2 | CGC GCA TGG TGG ATT CAC AAT CC |  |  | Tate & Simpson, 2003 |
|  |  |  |  |  |
| rpl32-F | GCA AGG ATA TTT GGC GGC TTT | Plastid *rpl32*-*trnL* |  | designed here |
| trnL-R | GAT GCT CTA GCA CTG CTT CCT |  |  |  |
| Leaf nodule symbionts | | | | |
| 16SB | AGA GTT TGA TCC TGG CTC AG | type1 and type 2A *rrn* operon (including 16S rRNA, ITS, and 23S rRNA) | forward | Van Oevelen et al. 2002 |
| 16S19 | AAC GGC AGC ACG GGA G |  | forward | Ku & Hu, 2014 |
| 16S21 | ACC CCC ACC TTT CAG CAG |  | reverse | Ku & Hu, 2014 |
| 16S22 | GTA GTC CAC GCC CTA AAC GA |  | forward | designed here |
| 23S4 | CAT CCA CCA CAT GCA CTT GT |  | reverse | designed here |
| 16S3 | TCG GTT CGA TCC CGT CAT CC |  | forward | Ku & Hu, 2014 |
| 23S2 | CTA CCT AAA TAG TTT TCG GAG AG |  | reverse | Ku & Hu, 2014 |
