# Supplementary material for: Molecular phylogeny of Asian Ardisia (Myrsinoideae, Primulaceae) and their leaf-nodulated endosymbionts, Burkholderia s.l. (Burkholderiaceae)
Source: PLoS One. 2022 Jan 19;17(1):e0261188. doi: 10.1371/journal.pone.0261188 (PMC8769342; doi:10.1371/journal.pone.0261188)
Supplement: S3 Table — (DOCX) [file pone.0261188.s008.docx]

**S3 Table. Detailed information of the *Burkholderia s.l.* sequences used in this study.**

| Taxon | Synonym | Strain | Accession number | Note |
| --- | --- | --- | --- | --- |
| *Burkholderia cepacia* |  | ATCC 25416 | CP012981.1 | chromosome 1 |
| *Burkholderia cenocepacia* |  | VC7848 | CP019668.1 |  |
| *Burkholderia gladioli* |  | ATCC 10248 | CP009323.1 | chromosome 1 |
| *Burkholderia glathei* | *Caballeronia glathei, Paraburkholderia glathei* | LMG 14190 | U96935.1 |  |
| *Burkholderia insecticola* |  | RPE64 | AP013059.1 | chromosome 2 |
| *Burkholderia oklahomensis* |  | C6786 | CP013359.1 | chromosome 2 |
| *Burkholderia pseudomallei* |  | BPs110 | CP036452.1 | chromosome 1 |
| *Burkholderia pyrrocinia* |  | mHSR5 | CP024903.1 | chromosome mHSR5_B |
| *Burkholderia rhizoxinica* | *Paraburkholderia rhizoxinica, Mycetohabitans rhizoxinica* | HKI 454, isolate B1 | FR687359.1 |  |
| *Burkholderia sordidicola* | *Caballeronia sordidicola, Paraburkholderia sordidicola* | SF-E2 | LC008478.1 |  |
| *Burkholderia sordidicola* | *Caballeronia sordidicola, Paraburkholderia sordidicola* | isolate Jm120 | DQ256491.1 |  |
| *Burkholderia symbiotica* | *Paraburkholderia symbiotica, Trinickia symbiotica* | JPY-347 | HM357232.1 |  |
| *Burkholderia telluris* | *Caballeronia telluris, Paraburkholderia telluris* | LMG 22936T (= CCUG 63060 = R-23326 = A57-4) | HE981727.1 |  |
| *Burkholderia thailandensis* |  | FDAARGOS_426 | CP023499.1 | chromosome 1 |
| *Burkholderia* sp. |  | PAMC 26561 | CP014306.1 | chromosome 1 |
| *Burkholderia* sp. |  | PAMC 28687 | CP014505.1 | chromosome 1 |
| *Burkholderia* sp. |  | BDU8 | CP013389.1 | chromosome 1 |
| *Burkholderia* sp. |  | MSMB0852 | CP013424.1 | chromosome 1 |
| *Burkholderia* sp. | *Burkholderia cordobensis* | RPE67 | AP014576.1 | chromosome 1 |
| *Burkholderia* sp. | *Burkholderia cordobensis* | YI23 | CP003088.1 | chromosome 2 |
| *Burkholderia* sp. |  | OLGA172 | CP014579.1 | chromosome 2 |
| *Burkholderia* sp. |  | MAK1 | KU195413.1 |  |
| *Burkholderia* sp. |  | isolate DF4EH10 | AJ884802.1 |  |
| *Burkholderia* sp. |  | SF-A1 | LC008480.1 |  |
| *Candidatus* Burkholderia hispidae | *Candidatus* Paraburkholderia hispidae | OL732, clone 4 | HQ849240.1 |  |
| *Candidatus* Burkholderia kirkii | *Candidatus* Paraburkholderia kirkii | 835462 | AF475068.1 |  |
| *Candidatus* Burkholderia nigropunctata | *Candidatus* Paraburkholderia nigropunctata | 19750521 | AY277698.1 |  |
| *Candidatus* Burkholderia rigidae | *Candidatus* Paraburkholderia rigidae | OL694, clone 1 | HQ849249.1 |  |
| *Candidatus* Burkholderia schumannianae | *Candidatus* Paraburkholderia schumannianae | SD1099, clone 5 | HQ849259.1 |  |
| *Caballeronia arationis* | *Burkholderia arationis* | LMG 29324 | NZ_FCOG02000237 |  |
| *Caballeronia concitans* | *Burkholderia concitans* | LMG 29315 | NR_145603.1 |  |
| *Caballeronia fortuita* | *Burkholderia fortuita* | LMG 29320 | NR_145600.1 |  |
| *Caballeronia glathei* | *Burkholderia glathei, Paraburkholderia glathei* | N15 | NR_037065.2 |  |
| *Caballeronia grimmiae* | *Burkholderia grimmiae, Paraburkholderia grimmiae* | GJ5 | MG270178.1 |  |
| *Caballeronia ptereochthonis* |  | LMG 29326 | NR_145596.1 |  |
| *Caballeronia terrestris* | *Burkholderia terrestris, Paraburkholderia terrestris* | R-23321 (= A13-11R-23321) | NR_125558.1 |  |
| *Caballeronia turbans* | *Burkholderia turbans* | LMG 29316 | NR_145604.1 |  |
| *Caballeronia udeis* | *Burkholderia udeis, Paraburkholderia udeis* | HMF7531 | MK123431.1 |  |
| *Caballeronia udeis* | *Burkholderia udeis, Paraburkholderia udeis* | LMG 27134 | NZ_FCOK02000155.1 |  |
| *Paraburkholderia aromaticivorans* |  | BN5 | CP022990.1 | chromosome 2 |
| *Paraburkholderia caryophylli* | *Burkholderia caryophylli, Trinickia caryophylli* | HAMBI 2159 | LT899950.1 |  |
| *Paraburkholderia endofungorum* | *Burkholderia endofungorum, Mycetohabitans endofungorum* | HKI 456 | NR_042584.1 |  |
| *Paraburkholderia fungorum* | *Burkholderia fungorum* | ATCC BAA-463 | CP010026.1 | chromosome 1 |
| *Paraburkholderia hospita* | *Burkholderia hospita* | DSM 17164 | CP026105.1 | chromosome 1 |
| *Paraburkholderia phenazinium* | *Burkholderia phenazinium* | A 1 (=LMG 2247) | NR_029212.1 |  |
