# Supplementary material for: Molecular phylogeny of Asian Ardisia (Myrsinoideae, Primulaceae) and their leaf-nodulated endosymbionts, Burkholderia s.l. (Burkholderiaceae)
Source: PLoS One. 2022 Jan 19;17(1):e0261188. doi: 10.1371/journal.pone.0261188 (PMC8769342; doi:10.1371/journal.pone.0261188)
Supplement: S4 Table — (DOCX) [file pone.0261188.s009.docx]

**S4 Table. Statistic values of comparing congruence within subtrees in TreeMap analysis.**

| Node | *Z*-value | *P*-value |  | Node | *Z*-value | *P*-value |
| --- | --- | --- | --- | --- | --- | --- |
| h0** | *Z* = 0.59462434 | *P* = 0.00000 [0.00000, 0.00242] |  | p0** | *Z* = 0.59462434 | *P* = 0.00000 [0.00000, 0.00242] |
| h1** | *Z* = 0.86720777 | *P* = 0.00000 [0.00000, 0.00242] |  | p1** | *Z* = 0.8430379 | *P* = 0.00000 [0.00000, 0.00242] |
| h3** | *Z* = 0.72424245 | *P* = 0.04500 [0.03249, 0.05847] |  | p2** | *Z* = 0.8450242 | *P* = 0.00000 [0.00000, 0.00242] |
| h5 | *Z* = 1.0 | *P* = 0.10100 [0.08270, 0.12024] |  | p3** | *Z* = 0.8954285 | *P* = 0.00000 [0.00000, 0.00242] |
| h7** | *Z* = 0.5880595 | *P* = 0.00000 [0.00000, 0.00242] |  | p4** | *Z* = 0.88982683 | *P* = 0.00000 [0.00000, 0.00242] |
| h8** | *Z* = 0.58429784 | *P* = 0.00000 [0.00000, 0.00242] |  | p5** | *Z* = 0.8620224 | *P* = 0.00000 [0.00000, 0.00242] |
| h9 | *Z* = 1.0 | *P* = 0.10900 [0.09006, 0.12887] |  | p6** | *Z* = 0.9002463 | *P* = 0.00000 [0.00000, 0.00242] |
| h11** | *Z* = 0.6140679 | *P* = 0.00000 [0.00000, 0.00242] |  | p7** | *Z* = 0.72424245 | *P* = 0.00500 [0.00072, 0.01027] |
| h12** | *Z* = 0.65517944 | *P* = 0.00000 [0.00000, 0.00242] |  | p8 | *Z* = 0.14285715 | *P* = 0.44900 [0.41850, 0.48026] |
| h13** | *Z* = 0.8579105 | *P* = 0.00000 [0.00000, 0.00242] |  | p9 | *Z* = 1.0 | *P* = 0.35000 [0.32079, 0.38002] |
| h14** | *Z* = 0.8430379 | *P* = 0.00000 [0.00000, 0.00242] |  | p11 | *Z* = 1.0 | *P* = 0.33700 [0.30805, 0.36676] |
| h15** | *Z* = 0.8475182 | *P* = 0.00000 [0.00000, 0.00242] |  | p14** | *Z* = 0.86720777 | *P* = 0.00100 [0.00000, 0.00424] |
| h16** | *Z* = 0.8800542 | *P* = 0.00000 [0.00000, 0.00242] |  | p15** | *Z* = 0.78839284 | *P* = 0.02400 [0.01481, 0.03416] |
| h17** | *Z* = 0.8763477 | *P* = 0.00000 [0.00000, 0.00242] |  | p17 | *Z* = 0.14285715 | *P* = 0.33900 [0.31001, 0.36880] |
| h18** | *Z* = 0.8620224 | *P* = 0.00000 [0.00000, 0.00242] |  | p18 | *Z* = 1.0 | *P* = 0.34600 [0.31687, 0.37594] |
| h19** | *Z* = 0.9002463 | *P* = 0.00000 [0.00000, 0.00242] |  | p21** | *Z* = 0.67304194 | *P* = 0.00000 [0.00000, 0.00242] |
| h20** | *Z* = 0.78839284 | *P* = 0.01600 [0.00848, 0.02450] |  | p22** | *Z* = 0.72424245 | *P* = 0.03599 [0.02478, 0.04819] |
| h21 | *Z* = 0.14285715 | *P* = 0.42100 [0.39073, 0.45204] |  | p23 | *Z* = 0.14285715 | *P* = 0.39600 [0.36603, 0.42676] |
| h22 | *Z* = 1.0 | *P* = 0.29600 [0.26807, 0.32477] |  | p24 | *Z* = 1.0 | *P* = 0.35300 [0.32373, 0.38308] |
| h27** | *Z* = 0.72424245 | *P* = 0.04600 [0.03336, 0.05960] |  | p26** | *Z* = 0.67409605 | *P* = 0.00000 [0.00000, 0.00242] |
| h29 | *Z* = 1.0 | *P* = 0.13000 [0.10953, 0.15139] |  | p27** | *Z* = 0.83826536 | *P* = 0.00000 [0.00000, 0.00242] |
| h32 | *Z* = 1.0 | *P* = 0.15500 [0.13295, 0.17796] |  | p28** | *Z* = 0.72424245 | *P* = 0.00899 [0.00333, 0.01565] |
| h34** | *Z* = 0.8475182 | *P* = 0.00000 [0.00000, 0.00242] |  | p29 | *Z* = 0.14285715 | *P* = 0.42100 [0.39073, 0.45204] |
| h35** | *Z* = 0.72424245 | *P* = 0.03700 [0.02563, 0.04934] |  | p30 | *Z* = 1.0 | *P* = 0.31799 [0.28949, 0.34733] |
| h36 | *Z* = 0.14285715 | *P* = 0.49700 [0.46632, 0.52841] |  | p32** | *Z* = 0.8475182 | *P* = 0.00000 [0.00000, 0.00242] |
| h37 | *Z* = 1.0 | *P* = 0.22800 [0.20237, 0.25450] |  | p33** | *Z* = 0.85984087 | *P* = 0.00000 [0.00000, 0.00242] |
| h39** | *Z* = 0.9133335 | *P* = 0.00000 [0.00000, 0.00242] |  | p34** | *Z* = 0.8800542 | *P* = 0.00000 [0.00000, 0.00242] |
| h40 | *Z* = 1.0 | *P* = 0.24000 [0.21390, 0.26697] |  | p35** | *Z* = 0.8763477 | *P* = 0.00000 [0.00000, 0.00242] |
| h42** | *Z* = 0.8620224 | *P* = 0.00000 [0.00000, 0.00242] |  | p36** | *Z* = 0.78839284 | *P* = 0.02500 [0.01563, 0.03535] |
| h43** | *Z* = 0.8478121 | *P* = 0.00000 [0.00000, 0.00242] |  | p37 | *Z* = 0.14285715 | *P* = 0.39500 [0.36504, 0.42574] |
| h44* | *Z* = 1.0 | *P* = 0.10000 [0.08178, 0.11916] |  | p38** | *Z* = 1.0 | *P* = 0.00000 [0.00000, 0.00242] |
| h46** | *Z* = 0.78839284 | *P* = 0.00800 [0.00265, 0.01434] |  | p41** | *Z* = 0.8478121 | *P* = 0.00000 [0.00000, 0.00242] |
| h47 | *Z* = 1.0 | *P* = 0.11600 [0.09653, 0.13640] |  | p42 | *Z* = 0.14285715 | *P* = 0.37100 [0.34140, 0.40139] |
| h49 | *Z* = 1.0 | *P* = 0.10400 [0.08546, 0.12348] |  | p45* | *Z* = 0.72424245 | *P* = 0.06600 [0.05097, 0.08198] |
| h51** | *Z* = 0.9133335 | *P* = 0.00000 [0.00000, 0.00242] |  | p46 | *Z* = 0.14285715 | *P* = 0.46600 [0.43540, 0.49734] |
| h52** | *Z* = 0.8954285 | *P* = 0.00000 [0.00000, 0.00242] |  | p47 | *Z* = 1.0 | *P* = 0.34700 [0.31785, 0.37696] |
| h53** | *Z* = 0.88982683 | *P* = 0.00000 [0.00000, 0.00242] |  | p49** | *Z* = 0.8763477 | *P* = 0.00000 [0.00000, 0.00242] |
| h54** | *Z* = 0.8620224 | *P* = 0.00000 [0.00000, 0.00242] |  | p50 | *Z* = 0.14285715 | *P* = 0.38400 [0.35420, 0.41459] |
| h56** | *Z* = 0.9002463 | *P* = 0.00000 [0.00000, 0.00242] |  | p51 | *Z* = 1.0 | *P* = 0.34300 [0.31393, 0.37288] |
| h58** | *Z* = 0.78839284 | *P* = 0.00200 [0.00000, 0.00586] |  | p53** | *Z* = 0.88982683 | *P* = 0.00000 [0.00000, 0.00242] |
| h59** | *Z* = 0.72424245 | *P* = 0.04200 [0.02991, 0.05506] |  | p54** | *Z* = 0.8620224 | *P* = 0.00000 [0.00000, 0.00242] |
| h60 | *Z* = 0.14285715 | *P* = 0.43500 [0.40460, 0.46616] |  | p55 | *Z* = 0.72424245 | *P* = 0.14399 [0.12262, 0.16630] |
|  |  |  |  | p56 | *Z* = 0.14285715 | *P* = 0.33300 [0.30414, 0.36267] |
|  |  |  |  | p57 | *Z* = 1.0 | *P* = 0.33000 [0.30121, 0.35961] |
|  |  |  |  | p59** | *Z* = 0.72424245 | *P* = 0.01100 [0.00475, 0.01823] |
|  |  |  |  | p60 | *Z* = 0.14285715 | *P* = 0.56400 [0.53356, 0.59514] |

*Z*-value, *P-*values and it’s 95% confidence level of each node are shown. The left half is the node of symbionts phylogeny and the right half is from the hosts. **P* < 0.1; ***P* < 0.05
